# Hobbyist preferences for pet freshwater turtles

**DOI:** 10.1111/cobi.70171

**Published:** 2025-11-14

**Authors:** Jingjing Zhao, Zhan Chen, Beilu Duan, Wuji Zheng, Anita Kar Yan Wan, Xiaoxi Zhang, Lishu Li, Tien Ming Lee

**Affiliations:** ^1^ School of Ecology Sun Yat‐sen University Shenzhen China; ^2^ School of Life Sciences Sun Yat‐sen University Guangzhou China; ^3^ Wildlife Conservation Society Beijing China; ^4^ Oxford Martin School University of Oxford Oxford UK

**Keywords:** consumer behavior, consumer segmentation, illegal wildlife trade, stated preference, trader certification, wild population trends, 消费者行为, 消费者细分, 非法野生动物贸易, 陈述性偏好, 商家证件, 野生种群趋势​, certificación comercial, comportamiento del consumidor, mercado de mascotas, mercado ilegal de fauna, preferencias declaradas, segmentación del consumidor, tendencias poblacionales silvestres

## Abstract

The burgeoning pet trade is a primary threat to wild freshwater turtles worldwide. Although the risks from commercial exploitation of turtles have been discussed widely, there is little empirical research on preferences for pet turtles from a hobbyists’ perspective. We conducted an online survey of nearly 2000 turtle hobbyists recruited via discussion forums in China. We collected information on turtle‐keeping experiences, past purchase behaviors, knowledge about the protection status of certain endangered species, and awareness of legality in the trade. We framed discrete choice experiments around different hypothetical scenarios about wild population statuses and captive‐breeding market conditions. We then used a random parameter logit model and a latent class logit model to identify key influencing factors and to explore the level of heterogeneity among turtle hobbyists. Consumers preferred inexpensive turtles, captive‐bred turtles for which traders’ certificates (e.g., business licenses, breeding permits, and proof of legal species origin) were available, online purchase, and delivery by the postal service. They were especially willing to buy turtles for which captive‐breeding techniques were well developed and turtles that had wild populations that were stable or increasing. Preferences were sometimes affected by social factors; for example, some consumers more willingly accepted high‐priced turtles under social influence, rather than when such factors were absent. We identified 3 distinct types of turtle consumers with heterogeneous preferences: 40.3% preferred cheap turtles, 31.1% preferred captive‐bred turtles, and 28.6% preferred both captive‐bred and inexpensive species. Overall, our results can inform interventions and campaigns to conserve freshwater turtles by targeting hobbyists with specific profiles.

## Resumen

El creciente mercado de mascotas es una de las principales amenazas para las tortugas silvestres de agua dulce en todo el mundo. Aunque se ha debatido ampliamente sobre los riesgos que conlleva la explotación comercial de las tortugas, hay pocos estudios empíricos sobre las preferencias de los aficionados en cuanto a las tortugas como mascotas. Realizamos una encuesta en línea a casi 2000 aficionados a las tortugas reclutados a través de foros de debate en China. Recopilamos información sobre sus experiencias en el cuidado de tortugas, sus comportamientos de compra en el pasado, sus conocimientos sobre el estado de protección de ciertas especies en peligro de extinción y su conciencia sobre la legalidad del comercio. Diseñamos experimentos de elección discreta en torno a diferentes escenarios hipotéticos sobre el estado de las poblaciones silvestres y las condiciones del mercado de cría en cautividad. A continuación, utilizamos un modelo logístico de parámetros aleatorios y un modelo logístico de clases latentes para identificar los factores clave que influyen y explorar el nivel de heterogeneidad entre los aficionados a las tortugas. Los consumidores prefirieron tortugas baratas, tortugas criadas en cautiverio para las que se disponía de certificados de los comerciantes (por ejemplo, licencias comerciales, permisos de cría y pruebas del origen legal de las especies), la compra en línea y la entrega por correo postal. Hubo una disposición especial a comprar tortugas para las que se habían desarrollado técnicas de crianza en cautiverio y tortugas cuyas poblaciones silvestres eran estables o estaban aumentando. En ocasiones, los factores sociales afectaron las preferencias; por ejemplo, algunos consumidores aceptaban más fácilmente las tortugas de alto precio bajo presión social, en comparación con cuando no existían dichos factores. Identificamos tres tipos distintos de consumidores de tortugas con preferencias heterogéneas: el 40.3 % prefirió las tortugas baratas, el 31.1 % prefirió las tortugas criadas en cautiverio y el 28.6 % prefirió tanto las especies criadas en cautiverio como las baratas. En general, nuestros resultados pueden servir de base para intervenciones y campañas de conservación de las tortugas de agua dulce dirigidas a aficionados con perfiles específicos.

## 摘要

当前快速扩张的宠物贸易已成为威胁全球野生淡水龟生存的主要因素之一。尽管学界对龟类商业性开发风险已有较多探讨, 但从宠物龟爱好者视角系统分析其偏好的实证研究仍十分匮乏。本研究通过中国境内龟类论坛招募近2000名爱好者, 开展线上问卷调查, 收集其养龟经历、购买行为及对部分濒危龟类保护现状和贸易合法性的认知, 并在此基础上设计涉及野生种群状况与人工繁育市场条件的假设情境开展离散选择实验, 结合随机参数Logit模型与潜在类别Logit模型识别关键影响因素并揭示偏好异质性。结果表明, 消费者偏好价格低廉、具备经营许可证、繁育许可证及合法来源证明的人工繁育个体, 以及可线上购买并邮寄的龟类, 其中人工繁育技术成熟的物种和野生种群稳定或增长的物种更受青睐。社会因素在特定情境下也显著影响偏好, 如部分消费者在社会因素作用下愿意接受高价龟种, 而缺乏此类因素时则表现出价格敏感性。进一步分析表明, 40.3%消费者偏好低价龟, 31.1%偏好人工繁育龟, 28.6%同时偏好人工繁育与低价龟。综上, 本研究结果可为制定面向不同偏好群体的差异化保护干预策略与宣传方案提供科学依据, 从而为淡水龟保护实践提供参考。

题目:宠物 淡水龟爱好者的购买偏好

## INTRODUCTION

Trade in reptiles is burgeoning worldwide and has led to hundreds of wild species being threatened (Marshall et al., 2020; Valdez, 2021). Freshwater turtles are now some of the most commonly traded species (Luiselli et al., 2016; Sigouin et al., 2017). At least 139 species of tortoises and freshwater turtles are listed under the Convention on International Trade in Endangered Species of Wild Fauna and Flora (CITES), meaning their international trade is subject to regulatory measures (CITES, 2010). However, CITES efforts to regulate wildlife often overlook lower value species, which may result in a lack of regulation for certain endangered or range‐restricted turtle species (Marshall et al., 2020). The extensive international trade in tortoises and freshwater turtles, whether arising from regulated species leading to illegal trade or unregulated species resulting in unsustainable practices, has driven lucrative poaching and cross‐border smuggling activities (Chen et al., 2009; Mendiratta et al., 2017; UNODC, 2020).

Since 1999, China has emerged as one of the largest consumers of freshwater turtles in the world, and international trade has been cited as the greatest threat to Asian turtles (Gong et al., 2009; van Dijk et al., 2000; Zhou & Jiang, 2008). In China, freshwater turtles are in demand not only for food and traditional medicine but also as pets, owing to turtles being revered by some Chinese as symbols of longevity, tenacity, and good fortune (Cheung & Dudgeon, 2006; Gong et al., 2009). To better conserve wild species of freshwater turtles, the Chinese government enacted the Wild Animal Conservation Law and banned the sales of precious and endangered (List of National Key Protected Animals) wild freshwater turtle species (Chen et al., 2023; The National People's Congress of the People's Republic of China, 2023). Species on the list can be traded if they are captive bred (The National People's Congress of the People's Republic of China, 2023).

The difficulty in distinguishing the provenance of these species presents a significant challenge for law enforcement personnel and increases the risk of protected species being laundered as captive‐bred species (Hughes et al., 2023; Tensen, 2016). Thus, some wild endangered freshwater species are still sold openly, even over mainstream internet sites (Hu, Lee, et al., 2022). To manage the captive breeding and sale of freshwater turtles, China plans to introduce a certification system for turtle breeding farms and a special identification system for management of individual captive‐bred turtles, but its current status remains unclear (Hong et al., 2022; Ministry of Agriculture & Rural Affairs of the People's Republic of China, 2021). Because law enforcement and policy implementation do not always fully mitigate threats to species’ survival, some measures have inadvertently driven markets underground (Gómez‐Rodríguez & Wilson, 2022). Therefore, supplementary market‐based approaches should be considered, with an emphasis on the trade‐offs between supply and demand in the market to enhance the effectiveness of these strategies (Cramer & Kittinger, 2021).

Theoretically, flooding the market with captive‐bred individuals could increase market supply and protect overexploited wild species, especially when the market is supply dominated (Davies et al., 2024; Sigouin et al., 2017). Yet, merely increasing the supply of captive‐bred wildlife may inadvertently stimulate latent demand and normalize purchases, potentially driving up the demand for target wild species beyond what captive‐breeding farms can supply (Davies et al., 2024; Tensen, 2016). Whether the legal products serve as substitutes depends on whether consumers have a preference for wild‐caught animals (Tensen, 2016). To effectively supplement regulatory measures, conservation practitioners are increasingly adopting innovative strategies to reduce demand for unsustainable wildlife products (MacFarlane et al., 2022; Naito et al., 2024; Thomas‐Walters et al., 2020; Veríssimo & Wan, 2019). Although mainstream conservation efforts are now beginning to use consumer data and insights from behavioral science to inform campaigns (Hu et al., 2024; Olmedo et al., 2018; Veríssimo et al., 2020), behavioral interventions often overlook the heterogeneous effects in a population with diverse preferences.

Pet freshwater turtle hobbyists, particularly because their preferences can significantly shape market dynamics, have attracted growing attention in the field of conservation (Hausmann, Cortés‐Capano, Fraser, et al., 2023; Moorhouse et al., 2017; Naito et al., 2024). Hobbyists are defined as individuals who actively engage in the collection, care, and breeding of species for personal interest rather than for commercial purposes and who play an important role in the pet trade (Pountney, 2023). A survey of pet markets in southern China estimated that the volume of freshwater turtles traded as pets could be as high as 1 million individuals of over 100 different species, which indicates the large scale of demand driven by hobbyists (Cheung & Dudgeon, 2006). Generally, hobbyists value turtles for their uniqueness, and sellers tend to have a larger proportion of juvenile animals because they are easier to keep as pets (Sigouin et al., 2017). Hobbyists specifically seek out species that are rare in the wild or scarce in the market (Hausmann, Cortés‐Capano, Fraser, et al., 2023). This may result in a cycle wherein increased exploitation reduces the population size, thus increasing a species’ value and ultimately leading to its extirpation (anthropogenic Allee effect) (Courchamp et al., 2006; Hausmann, Cortés‐Capano, & Di Minin, 2023). Hence, understanding how hobbyists make their choices, when given information regarding scarcity and rarity of pet freshwater turtles, is essential for developing targeted interventions aimed at promoting sustainable trade. Yet, a detailed exploration of how hobbyists weigh scarcity and rarity against other factors (e.g., purchasing channels, seller's certificate source, and price) when choosing species has not been conducted.

To rigorously investigate preferences, we used a discrete choice experiment (DCE). This is an effective tool that allows researchers to identify consumer preferences and what product attributes purchasers seek under certain purchase scenarios (Byun et al., 2018). DCEs are well known in economics and have been successfully adapted in studies of sustainable use (Natali et al., 2022; Zhao et al., 2023). We conducted a survey of Chinese freshwater turtle hobbyists with a DCE design. For pet turtle hobbyists, we sought to determine the effect on purchase choices of preferences for purchasing channels, seller's certificate, source, price, turtle wild population trends, and captive‐breeding techniques. We presented combinations of choice attributes and scenarios on a questionnaire. We aimed to provide insights for designing future conservation interventions for freshwater turtles and to improve market management.

## METHODS

### DCE and survey design

In the DCE part of the survey, respondents compared and weighed the specific choices according to different characteristics of their attributes and scenarios. We used qualitative and quantitative methods in the DCE. To gain relevant and current information on baseline conditions for our study area, we reviewed Chinese and English peer‐reviewed and gray literature published from 2001 to 2020 on consumer and market research; visited markets in Guangzhou and major exotic pet and pet turtle hobbyist conventions in Shanghai and Guangzhou, China; and interviewed pet turtle keepers, captive breeders, and policy makers from 2021 to 2022. Based on this work, we identified 4 attributes of hobbyists’ purchase choices: method of purchase (i.e., offline or online), seller's certificate (i.e., displayed or not), origin of individual (i.e., captive bred or wild caught), and prices. The prices used in the DCE were based on actual market prices for freshwater turtles observed during our visits and reflected real price variations. Choices were presented in the context of possible combination scenarios that broadly accounted for population trends of wild freshwater turtle species (i.e., increasing, stable, and decreasing) and captive‐breeding techniques (i.e., breeding in early development, where wild‐caught individuals are more prevalent, or well‐developed techniques, where captive‐bred individuals are more prevalent) (Appendix S1).

We used quantitative methods to test the selected DCE attributes, which helped us refine and finalize the final questionnaire. We used Ngene 1.0.1 (ChoiceMetrics) to produce a DCE design with 1000 Sobol draws from a normal distribution for each parameter prior (Bliemer et al., 2008). We selected the design with the lowest *D* error (0.1), which produced 45 alternatives and 15 choice sets. The final design included 18 choice sets that were randomly distributed among 3 blocks so that each block contained 6 choice sets (one choice set was repeated to test for consistency in choices and acted as one of the selection criteria for a valid response). Each choice set included a set of 2 combination scenarios with 2 available options for turtle type and a “neither of the above” option to opt out (Appendix S2). We tested the survey by using data on consumer preferences from a pilot survey of 418 respondents in multinomial logit (MNL) model used to estimate the characteristics of consumer choice preferences (Appendix S3). To design the final choice set efficiently, we incorporated the priors from the MNL model in a *D*‐efficient Bayesian design that followed the basic structure of the DCE (Dang Vu et al., 2022; Hinsley et al., 2022).

Our final questionnaire (Appendix S10) was in Chinese and had 3 major parts. The first part asked for informed consent (respondents were informed that the survey was anonymous and would not be traced back to them) and explained the topic and objective of the survey. After obtaining the written informed consent from participants, the survey commenced if respondents indicated they were >18 years old and kept turtles as pets.

The second part was about the respondent's experience with keeping freshwater turtles. For a deeper understanding of this, we asked whether respondents had ever raised or heard of the illegal sale of freshwater turtle species (i.e., determined exposure to illegal freshwater turtle species), had knowledge of what legal captive breeding is (knowledge of the definition of *captive bred*), had knowledge of the legality of trade in and protection statuses of freshwater turtle species (knowledge of freshwater turtle legality), and had family or friends who kept illegal freshwater turtle species (social influence) (detailed survey items in Appendix S10). We limited the list of freshwater turtle species to 7. Four species represented illegally traded species: Indo‐Chinese box turtle (*Cuora galbinifrons*, *Cuora bourreti*, or *Cuora picturata*), keeled box turtle (*Cuora mouhotii*), Southeast Asian box turtle (*Cuora amboinensis*), and big‐headed turtle (*Platysternon megacephalum*). They are critically endangered (CR) and endangered (EN) species as evaluated by IUCN and were characterized by underdeveloped captive‐breeding techniques, listing in CITES Appendices I and II, and primarily traded internationally as pets. For the remaining choices, we used 3 freshwater turtle species that are legally traded, are the most commonly found species in the market, are not included in the IUCN Red List and CITES, and are sourced legally through well‐established captive‐breeding practices (Appendix S4): snapping turtle (*Chelydra serpentina*), stink‐pot turtle (*Sternotherus odoratus*), and red‐eared slider (*Trachemys scripta elegans*) (Appendix S4).

**FIGURE 1:**
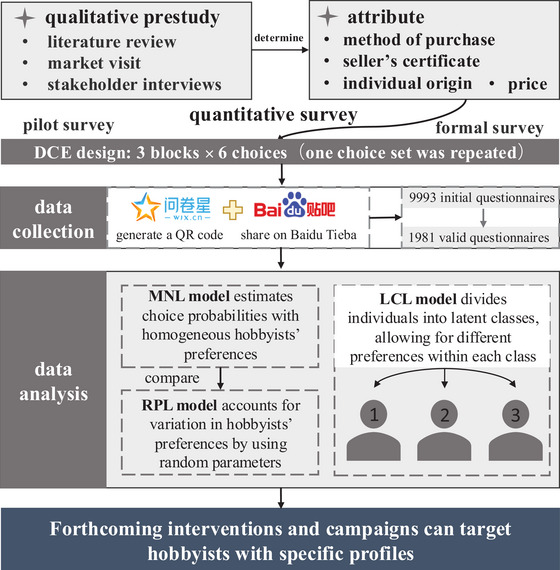
The framework and methodology flowchart of discrete choice experiment (DCE) to determine hobbyists’ preferences in pet freshwater turtles (MNL, multinomial logit model; RPL, random parameter logit model; LCL, latent class logit model).

The third section was the choice experiment (Figure 1). The last section contained sociodemographic questions on gender, residence in the last year (Appendix S5), employment status, education level, and personal annual income. Each respondent received a bonus payment of 15 CNY (∼US$2) on completion of the questionnaire.

### Data collection

This project is part of a larger project and was approved by the Wildlife Conservation Society Institution Review Board (20‐58RN). We conducted the pilot survey in June 2022 with an online sample panel with single‐access survey links on a local online survey platform (Wenjuanxing, WJX); an online distribution of links on turtle hobbyists’ discussion forums and community threads on Baidu Tieba (equivalent of Reddit); an offline sampling of visitors at a centralized flower, bird, and fish’ market in Guangzhou that is also a hotspot for trading pet turtles; and an offline random sampling of university students from Sun Yat‐sen University (Appendix S6).

The questionnaire was hosted on the WJX online survey platform, and a QR code was generated for the survey (Figure 1). This QR code was then shared on Baidu Tieba to collect data. This decision was supported by our preliminary interviews, which showed that a significant proportion of freshwater turtle hobbyists in China are young and often share their turtle‐keeping experiences on online forums, such as Baidu Tieba. We implemented the survey via an anonymous questionnaire distributed on Baidu Tieba in August 2022. Because our questionnaire provided a small monetary incentive, it attracted some respondents who were not freshwater turtle keepers and were only seeking the monetary reward. To ensure that only actual freshwater turtle keepers responded to our questionnaire, we used multiple quality control questions. At the beginning, respondents were asked a multiple‐choice question about which turtles they had raised. Later in the survey, they were prompted to write down the specific species of turtle they had owned but could not return to previous pages to check their earlier answers. This made it challenging for those who were not actual keepers to provide consistent responses (i.e., they needed to have a similar answer to 2 questions). We considered questionnaires with different answers invalid. A total of 9993 questionnaires were initially collected. Among these, 7662 respondents were not freshwater turtle keepers; thus, we retained 2331 responses. We excluded 31 questionnaires after filtering out incomplete or inattentive responses. We removed an additional 319 DCE responses in which the same choice set was repeated to test for consistency and the answers were inconsistent across the repeated choice sets, meaning these respondents failed the consistency check. Ultimately, we received 1981 valid questionnaires (data available upon request from T.L.).

### Data analyses

We used dummy coding with categorical variables due to their equivalence and lower likelihood of misinterpretation (Hu, Sun, et al., 2022). Each categorical variable was converted into a set of binary variables in which each category was represented as an individual variable and compared with other categories. We used an MNL model for the DCE (Figure 1). We assessed the aggregate preference heterogeneity of respondents with a random parameter logit (RPL) model (Figure 1). Relative to other discrete choice models, the RPL model relaxes the independence of irrelevant alternative property by allowing heterogeneity in hobbyists’ preferences (Train, 2003). Hence, we retained the RPL output as our main results. We assumed that attributes (including those in the scenarios) were normally distributed and the price had a lognormal distribution. We estimated 2 RPL models with 500 Halton draws (Train, 2003). We sequentially added the following variables as interactions in the RPL models: demographics (i.e., gender, age, annual income), exposure to illegal freshwater turtle species (i.e., high, medium, low), social influence on target turtle species (i.e., yes if family and friends had kept illegal freshwater turtles; no if they have not), score of turtle legality knowledge (i.e., higher score indicates greater awareness of legality), and score of captive‐bred concept (i.e., higher score indicates greater awareness of the concept of captive breeding) (Appendix S7). Based on the assessment of the minimum Akaike information criterion (AIC) and Bayesian information criterion (BIC) (Natali et al., 2022), we chose 4 RPL models as the best models.

Then, we estimated the willingness to pay (WTP) for all attributes, which was consumer willingness to pay for a freshwater turtle with a particular attribute level, holding other attribute levels constant (Mariel et al., 2021). The standard errors of WTP estimates were obtained using the delta method. The variable β_attribute_ was the estimated parameter of the attribute level, and μ and σ were the mean and standard deviation of the price parameter, respectively:

$$\mathrm{WTP}=\frac{\rho_{\mathrm{attribute}}}{\exp\left(\mu +\sigma^{2}/2\right)},$$

where ρ_attribute_​ is the estimated coefficient for the attribute of interest, and μ and σ are the mean and standard deviation of the price coefficient, respectively, based on an assumption of lognormal distribution.

We included the covariates in the latent class logit (LCL) model (Figure 1), which is considered one of the best approaches to determining heterogeneity in preferences and class membership (Shen, 2009). With the LCL model, we classified consumers by preferences, which helped us identify differences in their decision‐making. This segmentation should allow for more targeted interventions and tailoring of strategies to the specific needs of each consumer class. Each class was interpreted as representing a distinct turtle hobbyist type, which could be helpful in targeting interventions. To understand hobbyists’ characteristics in each group, we included these covariates and compared the main statistics across the LCL models for up to 5 classes. Based on the assessment of the minimum Akaike information criteria (AIC) and Bayesian information criteria (BIC) (Natali et al., 2022), we used a 3‐class model (Appendix S8).

The MNL, RPL, WTP, and LCL analyses were conducted using the APOLLO R package (Hess & Palma, 2019).

## RESULTS

The majority of hobbyists were men (73%) under the age of 35 (80%). Most respondents had full‐time jobs, had a medium or higher level of annual income, and lived in Guangdong Province, which accounted for 13.8% of the respondents (Table 1; Appendix S5). There were significant differences between the gender and education variables in our sample and those of the general Chinese population (Table 1).

**TABLE 1:** Sociodemographic information on respondents to a survey of pet freshwater turtle hobbyists in China (1981 survey respondents).

Variables	Option	Respondents (%)	China population (%)	*z* ^a^
Gender	Male	73.14	51.24	21.80^**^
	Female	26.85	48.76	−21.81^**^
Age	18–24	40.83		
	25–29	24.93		
	30–34	21.10		
	35–39	8.63		
	40–44	2.97		
	45–49	0.75		
	50–54	0.61		
	>55 years old	0.15		
Education	Elementary school or below	0.20	34.94	−21.81^**^
	Middle school	0.90	34.51	−29.25^**^
	High school or vocational school	11.40	15.09	−5.10^**^
	Junior college or university undergraduate and above	87.47	15.47	69.69^**^
Employment status	Full‐time employment	68.70		
	Part‐time employment	6.46		
	Housewife or husband	2.72		
	Student	13.52		
	Retired	0.30		
	Independent or freelance	7.62		
	Other	0.65		
Income	<¥10,000 (∼$0–$1400)	0.65		
	¥10,000–50,000 (∼$1400–$7000)	12.97		
	¥50,000–100,000 (∼$7000–$14,000)	13.88		
	¥100,000–150,000 (∼$14,000–$21,000)	23.82		
	¥100,000–150,000 (∼$14,000–$21,000)	26.55		
	¥150,000–200,000 (∼$21,000–$28,000)	13.37		
	¥200,000 and above (∼$28,000 and above)	9.38		

*Note*: China population  data from the National Bureau of Statistics of China (https://data.stats.gov.cn/easyquery.htm?cn=C01&zb=A0305&sj=2022).

^a^ Significance: *p < 0.05; **p < 0.01.

### Freshwater turtle hobbyist preferences

Respondent preferences were qualitatively similar with the MNL model (Appendix S9) and 4 RPL models (Table 2). However, we focused on the RPL model because it captures individual‐level preference heterogeneity, as indicated by significant standard deviation estimates for all attributes except population trends in the wild. Nearly 20% of respondents chose the opt‐out option, which had a significantly negative coefficient and thus suggested a general preference for purchasing a freshwater turtle over not purchasing one.

**TABLE 2:** Model estimates (random parameter logit) from a discrete choice experiment on consumer preferences for pet freshwater turtles in China (1981 survey respondents).

Attribute^a^	State	Model 1 No interactions (SE)^b^	Model 2 Interaction with exposure level (SE)^b^	Model3 Interaction with social influence (SE)^b^	Model4 Interaction with exposure level and social influence (SE)^b^
Population trends in the wild (ref. decreasing)	Increasing	0.82^**^ (0.11)	0.42^*^ (0.15)	0.43^**^ (0.10)	0.16 (0.21)
	Stable	0.23^**^ (0.10)	0.44^*^ (0.17)	0.35^**^ (0.13)	0.30 (0.26)
Captive‐breeding techniques (ref. early development)	Well developed	0.24^**^ (0.09)	0.06 (0.17)	0.33^**^ (0.12)	0.43 (0.25)
Method of purchase (ref. online, delivery by postal service)	Offline, physical store	−0.78^**^ (0.05)	−0.79^**^ (0.08)	−0.76^**^ (0.06)	−0.80^**^ (0.13)
Relevant seller certificates (ref. not displayed)	Displayed	0.74^**^ (0.06)	0.74^**^ (0.10)	0.81^**^ (0.08)	0.97^**^ (0.16)
Individual source (ref. wild caught)	Captive bred	0.24^**^ (0.09)	0.82^**^ (0.09)	0.93^**^ (0.07)	0.84^**^ (0.15)
Price (CNY/individual)		−1.01^**^ (0.05)	−0.63^**^ (0.07)	−1.12^**^ (0.06)	−0.87^**^ (0.11)
ASC		−3.61^**^ (0.17)	−3.48^**^ (0.17)	−3.46^**^ (0.17)	−3.53^**^ (0.17)
Standard deviation					
Population trends in the wild (ref. decreasing)	Increasing	0.18 (0.31)	0.03 (0.03)	0.03 (0.03)	0.04 (0.03)
	Stable	0.15 (0.82)	0.02^**^ (0.03)	0.02 (0.03)	−0.00 (0.03)
Captive‐breeding techniques (ref. early development)	Well developed	1.01^**^ (0.23)	0.93^**^ (0.26)	0.94^**^ (0.20)	−1.18^***^ (0.20)
Method of purchase (ref. online, delivery by postal service)	Offline, physical store	−0.91^**^ (0.07)	−1.21^**^ (0.08)	−0.89^**^ (0.07)	−0.94^**^ (0.08)
Relevant seller certificates (ref. not displayed)	Displayed	−1.22^**^ (0.08)	−1.21^**^ (0.08)	−1.22^**^ (0.08)	−1.25^**^ (0.08)
Individual source (ref. wild caught)	Captive bred	1.16^**^ (0.07)	1.16^**^ (0.07)	1.14^**^ (0.07)	1.15^**^ (0.07)
Price (CNY/individual)		−1.04^**^ (0.05)	−1.00^**^ (0.04)	−1.01^**^ (0.05)	−1.01^**^ (0.04)
Interaction					
Population trends in the wild increasing × high exposure (ref. low exposure)			−0.05 (0.19)		0.25 (0.25)
Population trends in the wild stable × high exposure (ref. low exposure)			−0.13 (0.23)		0.07 (0.30)
Captive‐breeding techniques × high exposure (ref. low exposure)			0.28 (0.21)		−0.02 (0.28)
Method of purchase × high exposure (ref. low exposure)			0.02 (0.11)		0.01 (0.15)
Relevant seller certificates × high exposure (ref. low exposure)			0.02 (0.14)		−0.19 (0.19)
Individual source × medium exposure (ref. high exposure)			0.09 (0.12)		0.09 (0.17)
Price × high exposure (ref. low exposure)			−0.44^**^ (0.99)		−0.22 (0.12)
Population trends in the wild increasing × medium exposure (ref. low exposure)			0.24 (0.18)		0.41^*^ (0.23)
Population trends in the wild stable × medium exposure (ref. low exposure)			0.00 (0.22)		0.11 (0.23)
Captive‐breeding techniques × medium exposure (ref. low exposure)			0.08 (0.19)		−0.05 (0.22)
Method of purchase × medium exposure (ref. low exposure)			0.06 (0.19)		0.06 (0.11)
Relevant seller certificates × medium exposure (ref. low exposure)			0.00 (0.12)		−0.10 (0.14)
Individual source × medium exposure (ref. low exposure)			0.16 (0.11)		0.16 (0.13)
Price × medium exposure (ref. low exposure)			−0.51^**^ (0.08)		−0.39^**^ (0.09)
Population trends in the wild increasing × social influence (ref. none)				0.21 (0.15)	0.33 (0.19)
Population trends in the wild stable × social influence (ref. none)				0.18 (0.18)	0.20 (0.23)
Captive‐breeding techniques × social influence (ref. none)				−0.38^**^ (0.16)	−0.33 (0.22)
Method of purchase × social influence (ref. none)				0.00 (0.08)	0.01 (0.12)
Relevant seller certificates × social influence (ref. none)				−0.15 (0.10)	−0.25 (0.15)
Individual source × social influence (ref. none)				−0.08 (0.10)	−0.02 (0.13)
Price × social influence (ref. none)				0.34^**^ (0.07)	0.25^**^ (0.09)
AIC		16,759.83	16,767.41	16,756.00	16,751.30
BIC		16,875.04	16,943.43	16,921.62	17,017.73
Log likelihood		−8363.92	−8353.71	−8355	−8338.65
Number of individuals	1981				
Number of rows	9905				

^a^ Definitions: high exposure, individuals who have raised illegal freshwater turtle species; medium exposure, individuals who haveheard of illegal freshwater turtle speciesbut have never raised one; low exposure, individuals who have neither raised nor heard of illegal freshwater turtle species; Ref., reference level; ASC, alternative specific constant (opt‐out or no‐purchase option); AIC, Akaike information criterion; BIC, Bayesian information criterion.

^b^ Significance: *p < 0.05; **p < 0.01.

All included attributes represented key dimensions of hobbyists’ preferences, but their levels of relative importance differed (Table 2). Price was one of the most important components; respondents preferred less expensive freshwater turtles. Across scenarios, respondents expressed a greater willingness to purchase freshwater turtles when wild populations were stable or increasing and when captive‐breeding techniques were well developed. Respondents were more inclined to choose captive‐bred individuals over wild‐caught individuals and preferred sellers who provided official certificates. Finally, online platforms with mail delivery were generally favored over offline physical stores.

With the RPL models, we found that the interaction covariates of exposure to illegal freshwater turtle species and social influence had significant modifying effects on the price of freshwater turtles (Table 2). Specifically, even at medium or high levels of exposure, respondents continued to prefer lower priced turtles. However, under the influence of social norms, preferences shifted to more expensive freshwater turtles. We found a significant negative interaction between social influence and well‐developed captive‐breeding techniques.

**FIGURE 2:**
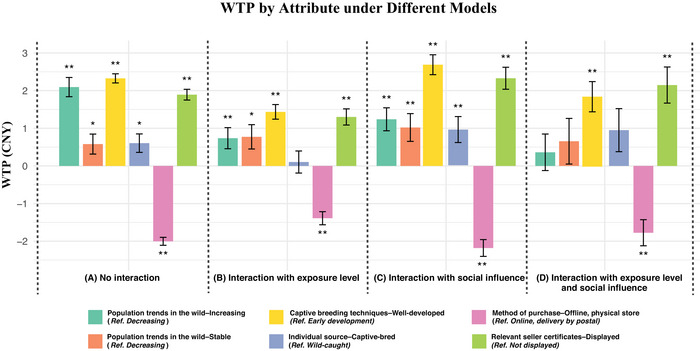
Estimates of the willingness to pay (WTP) of consumers (1981 survey respondents) for pet freshwater turtles in China based on random parameter logit (RPL) models: (a) baseline model without interaction terms, (b) model including interactions between choice attributes and respondents’ exposure level to illegal freshwater turtle species, (c) model including interactions between choice attributes and respondents’ social influence, and (d) model including interactions between choice attributes and respondents’ exposure level to illegal freshwater turtle species and social influence (bars, WTP values for different attribute levels relative to their respective reference [ref.] levels; whiskers, SE; *p < 0.05; **p < 0.01).

This pattern was further reflected in the WTP estimates derived from the RPL models (Figure 2). Across all 4 model specifications, respondents consistently showed positive WTP for well‐developed captive‐breeding techniques and displayed seller certificates, though effect sizes varied slightly depending on interaction terms. The WTP for purchasing from offline physical stores was negative in all models (range −1.39 CNY to −2.18 CNY), indicating that respondents were willing to pay 1.39–2.18 CNY more for online purchases with delivery than for buying from a physical store.

### Latent class segments of freshwater turtle hobbyists

**FIGURE 3:**
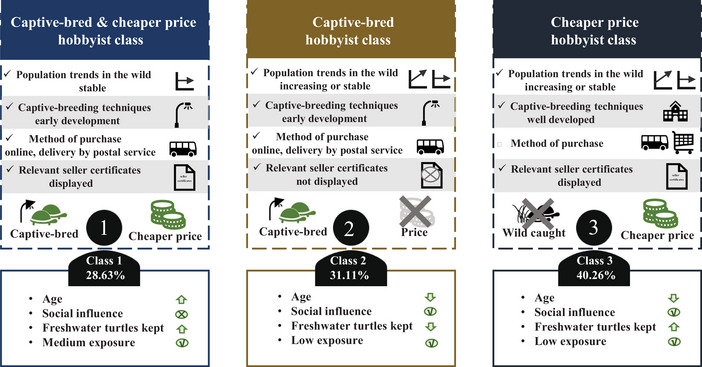
Model estimates from the discrete choice experiment on consumer preferences (1981 survey respondents) for pet freshwater turtles in China (upward‐pointing arrows, positive correlation between the continuous variable and preference; downward‐pointing arrows, negative correlation; checkmarks in a circle, category positively influences preference; ×, negative influence on preference; checkmarks, attribute level affects consumer preferences, × in a circle, attribute level does not affect consumer preferences).

The LCL model identified 3 distinct latent classes of freshwater turtle hobbyists based on their preferences (Figure 3; Table 3). The hobbyist classes were as follows. Class 1 (28.63% of the sample) comprised older hobbyists who were aware of endangered or illegal freshwater turtles and were less influenced by their social circles. They showed a strong preference for sellers who displayed certificates and favored purchasing captive‐bred, lower‐priced freshwater turtles via online delivery, particularly when breeding techniques were still developing and wild populations were stable. Class 2 (31.11%) included younger hobbyists who had kept freshwater turtles before and were easily influenced by their social circles. They tended to disregard whether certificates were displayed but still preferred captive‐bred turtles delivered online, especially when breeding techniques were developing and wild populations were stable or increasing. Class 3 (40.26%) consisted mainly of younger hobbyists who were more easily influenced by their social circles. They had kept freshwater turtles but had never heard of or kept any endangered or illegal freshwater turtles. They preferred to buy cheaper freshwater turtles when captive‐breeding techniques were well developed and their wild populations were stable or increasing.

**TABLE 3:** Model estimates (latent class logit model) from a discrete choice experiment on consumer preferences for pet freshwater turtles in China (1981 survey respondents), including standard errors in parentheses.

Attribute^a^	State	Class 1 Captive bred and cheap price (SE)^b^	Class 2 Captive bred (SE)^b^	Class 3 Cheap price (SE)^b^
Population trends in the wild (ref. decreasing)	Increasing	0.10 (0.11)	0.99^*^ (0.36)	1.49^**^ (0.44)
	Stable	0.55^**^ (0.17)	1.04^*^ (0.39)	1.57^**^ (0.46)
Captive‐breeding techniques (ref. early development)	Well developed	−0.24^*^ (0.12)	−1.62^**^ (0.41)	1.99^**^ (0.59)
Method of purchase (ref. online, delivery by postal service)	Offline, physical store	−1.05^**^ (0.16)	−4.96^**^ (1.17)	−0.01 (0.05)
Relevant seller certificates (ref. not displayed)	Displayed	1.35^**^ (0.14)	−0.90^**^ (0.14)	0.70^**^ (0.06)
Individual source (ref. wild caught)	Captive bred	1.09^**^ (0.19)	5.67^**^ (1.17)	−0.06 (0.05)
Price per individual		−1.49^**^ (0.09)	0.07 (0.07)	−0.51^**^ (0.05)
ASC		−1.95^**^ (0.14)		
Covariate				
Age		0.18^**^ (0.06)	0.08 (0.06)	
Gender (ref. female)		−0.14 (0.15)	0.18 (0.15)	
Annual income		−0.09 (0.05)	−0.02 (0.05)	
Number of freshwater turtles kept		−0.04 (0.09)	−0.22^*^ (0.10)	
Breeding year		0.01 (0.08)	0.07 (0.08)	
High exposure (ref. low exposure)		0.24 (0.24)	0.09 (0.23)	
Medium exposure (ref. low exposure)		0.51^*^ (0.19)	0.04 (0.18)	
Social influence (ref. none)		−0.48^*^ (0.18)	−0.11 (0.18)	
Score of captive‐bred definition knowledge		0.21 (0.13)	0.14 (0.13)	
Score of freshwater turtle legality knowledge		0.04 (0.14)	0.19 (0.14)	
AIC	16,565.73			
BIC	16,889.77			
Log likelihood	−10,881.75			
Number of individuals	1981			
Number of rows	9905			

^a^ Definitions: high exposure, individuals who have raised illegal freshwater turtle species; medium exposure, individuals who haveheard of illegal freshwater turtle species but have never raised one; low exposure, individuals who have neither raised nor heard of illegal freshwater turtle species; Ref., reference level; ASC, alternative specific constant (opt‐out or no‐purchase option); AIC, Akaike information criterion; BIC, Bayesian information criterion.

^b^ Significance: *p < 0.05; **p < 0.01.

## DISCUSSION

### Interpreting freshwater turtle hobbyists’ preferences

We found that pet freshwater turtle hobbyists were more inclined to purchase a cheaper, captive‐bred freshwater turtle via online shops with postal delivery, especially in contexts where captive‐breeding techniques were well developed and wild turtle populations were stable or increasing (Table 2). Freshwater turtles widely traded in the pet market are generally categorized as either wild or captive bred (Luiselli et al., 2016). In markets where many species have well‐established captive‐breeding techniques and stable wild populations, hobbyists are better able to select pets that align with their preferences. Moreover, although hobbyists purchase species as pets, they aim to avoid negatively affecting the survival of species (Naito et al., 2024). Therefore, purchasing freshwater turtles in contexts with mature breeding techniques and abundant wild populations alleviates concerns regarding the species’ well‐being (Hausmann, Cortés‐Capano, Fraser, et al., 2023).

Consistently, positive WTP for captive‐bred turtles and stable or increasing wild populations further confirmed this conservation‐friendly preference (Figure 2). This finding is consistent with the results of other studies in which wild‐sourced species tend to be the least preferred option. Although wild‐sourced wildlife products are typically more expensive than farmed ones, the higher price of farmed freshwater turtles, compared with wild‐caught turtles, is due to their lower extinction risk, lower maintenance needs, and lower mortality rates (Hong et al., 2022). However, this preference may contradict consumers’ tendency to favor low‐priced products, which may result in wild species continuing to face significant trade risks when there is a lack of sufficiently affordable captive‐bred freshwater turtles in the market.

Social media platforms are playing an increasingly influential role in boosting the online wildlife trade by providing an easily accessible way to share information, advertise specimens, and arrange sales (Olden et al., 2021; UNODC, 2020; Ye et al., 2020). Similar to other wildlife product markets, trade in freshwater turtles is shifting from offline to online, which has become a major channel of pet sales (Liu et al., 2021). The prevalence of online pet trading makes the law enforcement and regulation fraught with new challenges (Bennett, 2011). For example, when online sales points are detected by law enforcement, traffickers simply switch platforms to avoid it (Olden et al., 2021).

However, freshwater turtle hobbyists’ preference for sellers who provide legal trading certificates can be seen as a promising finding amid the difficulties in regulating online sales. This is further supported by their consistently positive WTP for transactions involving displayed certificates, as shown across all model specifications (Figure 2). This preference is partly driven by a social norm of trusting documents issued by Chinese authorities, which are perceived as safeguards for ensuring transaction legitimacy. According to the Wildlife Protection Law and the List of National Key Protected Wild Animals, all sellers in China must obtain a license for the artificial breeding of such species (The National People's Congress of the People's Republic of China, 2023). Thus, enhancing consumer awareness of relevant legal certificates and strictly regulating the certificate issuance process may contribute to preventing consumers from inadvertently purchasing illegally traded endangered species from sellers to some extent. However, such licenses merely indicate whether the seller is permitted to conduct captive breeding, which can create opportunities for laundering illegally wild‐caught turtles (ADM Capital Foundation, 2018). In Hong Kong, for example, where there is no requirement for marking and identifying specimens, it is challenging to differentiate between a legally traded, captive‐bred freshwater turtle and an illegally sourced, wild‐caught golden coin turtle (*Cuora trifasciata*) (ADM Capital Foundation, 2018). Given this, and especially where captive‐breeding techniques remain underdeveloped, both wild‐sourced and captive‐bred individuals should be regulated in parallel, rather than focusing solely on the former. Therefore, targeted and practical interventions aimed at improving consumer understanding of trading certificate legitimacy are also crucial (Hu et al., 2024).

### Freshwater turtle hobbyist categories and implications for future interventions

We identified 3 distinct types of hobbyists (Figure 3) and, based on these categories, developed targeted strategies for future interventions to promote sustainable freshwater turtle trade.

Given their relatively older age and stable preferences, class 1 hobbyists tend to avoid social influences. For this group, interventions should focus on educating them about the potential negative impact of purchasing wild‐caught turtles on the markets and ecosystems (Naito et al., 2024). By framing the higher cost of captive‐bred turtles as an investment to promote sustainability and conservation, these hobbyists can be encouraged to prioritize sustainable options over cheaper, wild‐caught turtles, avoiding impulsive, price‐driven purchases (Natali et al., 2022). The key is to present the ecological and legal benefits of selecting sustainably farmed turtles and guide them toward more responsible, long‐term decisions (Cardoso et al., 2021).

Class 2 hobbyists, who are younger and more susceptible to peer influence, often lacked experience in keeping turtles, making them less likely to prioritize the verification of seller certificates. Educating them on the importance of verifying legal certificates before making a purchase is essential (Hu et al., 2024). Their preference for online shopping with postal delivery offers an opportunity for targeted intervention (Feddema et al., 2020). Online platforms could play a role in prompting them to verify the legitimacy of captive‐bred turtle claims and align their choices with species conservation goals. Given that they are less price sensitive, the intervention could also stress the long‐term environmental and legal risks of purchasing high‐priced endangered species, helping them make more informed and sustainable decisions (Naito et al., 2024).

Although class 3 hobbyists were typically younger and more impressionable, they also possessed previous experience in keeping turtles, which may lead to overconfidence in their ability to care for wild and captive‐bred species. Interventions for this group should aim to harness social influence and enhance their sense of responsibility as caretakers (Wallen & Daut, 2017). By showcasing examples and role models of experienced hobbyists who prioritize captive‐bred freshwater turtles, they may be encouraged to adopt more conservation‐focused practices (Moorhouse et al., 2017). Incorporating social influence and ecological responsibility would help guide them toward more sustainable and ethical purchasing habits (Naito et al., 2022).

Our study has several limitations. First, our sample was drawn primarily from younger and more educated individuals who tend to dominate online survey platforms, which may not fully represent the broader population of freshwater turtle hobbyists in China. Second, the relatively small WTP estimates may be partly due to the broad price range (100–3000 CNY) used in our DCE, which was based on real market prices across various freshwater turtle species. Because we focused on general categories rather than specific species, this wide range may have limited respondents’ sensitivity to price variation. Third, although our experiment has the potential to develop techniques for studying undesirable preferences, bias in choices with hypothetical situations cannot be avoided, particularly when addressing sensitive topics, such as illegal pet wildlife trade. Future research could address these limitations by adopting more representative sampling strategies, using narrower or species‐specific price ranges, and further refining methods to improve validity in sensitive contexts. In conclusion, our DCE evaluated hobbyists’ preferences for certain pet freshwater turtle attributes, and our insights could be used to understand consumer demand and help identify target audiences for future interventions.

## AUTHOR CONTRIBUTIONS

Jingjing Zhao, Zhan Chen, Anita Kar Yan Wan, Beilu Duan, Xiaoxi Zhang, Lishu Li, and Tien Ming Lee designed research. Zhan Chen, Beilu Duan, Wuji Zheng, Anita Kar Yan Wan, and Xiaoxi Zhang collected survey data. Jingjing Zhao analyzed the data. Jingjing Zhao and Zhan Chen wrote the initial draft, and all authors revised the manuscript.

## Supporting information



Supplementary Materials.
