# Supplementary material for: Hobbyist preferences for pet freshwater turtles
Source: Conserv Biol. 2025 Nov 14;40(2):e70171. doi: 10.1111/cobi.70171 (PMC13036315; doi:10.1111/cobi.70171)
Supplement: Supplementary file 1 — Supplementary Materials. [file COBI-40-e70171-s001.docx]

**Supporting Information**

**Disentangling hobbyists’ preferences for pet freshwater turtles: A discrete choice experiment in China**

Appendix S1. Choice experiment’s scenarios, attributes and their levels, and the hypotheses.

| ***Scenarios / Attributes*** | ***Description*** | ***Levels*** | ***Hypotheses*** |
| --- | --- | --- | --- |
| **Freshwater turtle’s wild population trends** | A freshwater turtle species’ wild population size is less than 2000 with the trend of decreasing, maintain stable or increasing | Decreasing, Stable, Increasing | Turtle’s wild population trends would affect pet turtle hobbyist preference of freshwater turtle species |
| **Captive breeding techniques** | A freshwater turtle species’ captive breeding techniques are in-development or well-developed, with either mostly wild-caught individuals or abundant captive-bred ones sold in the market | In early development, Well-developed (Mostly wild-caught, Abundant captive-bred) | Captive breeding techniques would affect pet turtle hobbyist preference of freshwater turtle species |
| **Methods of purchase** | Purchase and obtain freshwater turtle individuals from offline physical store or market, or online and obtained by *postal service* delivery | Offline physical pet store or market, online with delivery by postal service | Pet freshwater turtle hobbyist prefer to purchase turtles online by delivery mail over in offline store and collect in person |
| **Relevant sellers’ certificates** | Seller display or not display any potential certificates or legal documentation required for selling, breeding or approving source of freshwater turtles in sold | Not displayed, Displayed | Pet freshwater turtle keepers prefer to purchase turtles from sellers who display relevant certificates (legal source) over sellers do not display them (illegal source). |
| **Individual source** | Source (or provenance) of the freshwater turtle individuals: either caught from wild or bred in captive farms. | Wild-caught, Captive-bred | Pet freshwater turtle hobbyist prefer to purchase turtles from wild-caught over that are captive-bred |
| **Price (CNY/individual)** | Four levels of price for juvenile freshwater turtles: 100 CNY (~13.89 USD), 500 CNY (~69.46 USD), 1000 CNY (~138.93 USD), or 3000 CNY (~416.78 USD) | 100, 500, 1000, 3000 | Pet freshwater turtle hobbyist prefer to purchase turtles that are lower in price |

Appendix S2. An example of a choice set presented to the respondent

**
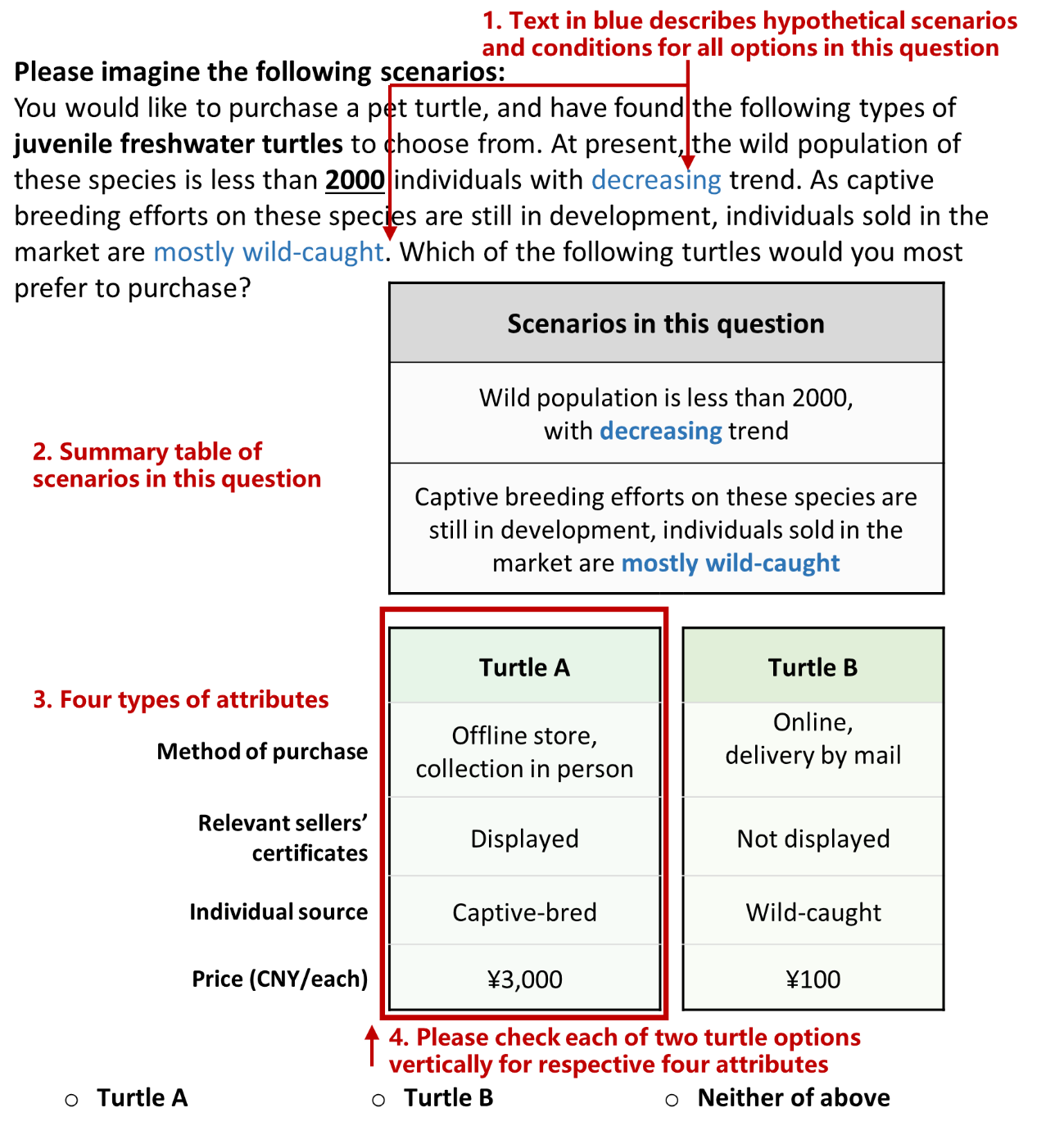
**

Appendix S3. Summary of the Multinominal Logit (MNL) estimates from the discrete choice experiment on consumer preferences for the pilot survey (N = 418), including standard errors in parentheses. Significance levels: **P* < 0.05, ***P* < 0.01.

| ***Attribute*** | ***Level*** | ***Estimate (sd)*** |  |
| --- | --- | --- | --- |
| Population trends in the wild  *(Ref. Decreasing)* | Increasing | 0.42**  (0.12) | |
|  | Stable | 0.19  (0.14) | |
| Captive breeding techniques  *(Ref. Early development)* | Well-developed | -0.32**  (0.12) | |
| Method of purchase  *(Ref. Online, delivery by mail)* | Offline, collection in-person | 0.17**  (0.06) | |
| Relevant seller certificates  *(Ref. Not displayed)* | Displayed | 0.96**  (0.08) | |
| Individual source  *(Ref. Wild-caught)* | Captive-bred | -0.58**  (0.08) | |
| Price (CNY /individual) |  | -0.15**  (0.04) | |
| ASC |  | -0.28*  (0.13) | |
| **AIC** | 16930.91 |  | |
| **BIC** | 17003.21 |  | |
| **Log-Likelihood** | -9231.69 |  | |
| **Number of individuals** | 418 |  | |
| **Number of rows** | 2090 |  | |

Appendix S4. The information of seven species

| ***Common species name*** | ***Scientific species name*** | ***Chinese name*** | ***IUCN*** | ***CITES*** | ***Trade type*** | ***Captive breeding technique*** |
| --- | --- | --- | --- | --- | --- | --- |
| Indochinese box turtle | *Cuora galbinifrons*  */Cuora bourreti*  */Cuora picturata* | 黄额闭壳龟 | CR | Appendix I | Pet, food | *Early development* |
| Keeled box turtle | *Cuora mouhotii* | 锯缘摄龟 | EN | Appendix II | Pet, food | *Early development* |
| Big-headed turtle | *Platysternon megacephalum* | 大头平胸龟 | CR | Appendix I | Pet | *Early development* |
| Southeast Asian box turtle | *Cuora amboinensis* | 马来闭壳龟 | EN | Appendix II | Pet, medicine | *Early development* |
| Snapping turtle | *Chelydra serpentina* | 鳄龟 | LC | Not listed | Pet | *Well-developed* |
| Stink-pot turtle | *Sternotherus odoratus* | 麝香龟 | LC | Not listed | Pet | *Well-developed* |
| Red-eared slider | *Trachemys scripta elegans* | 红耳龟 | LC | Not listed | Pet | *Well-developed* |

Appendix S5. Distribution map of proportion of hobbyists' residences, at the province level, across China.


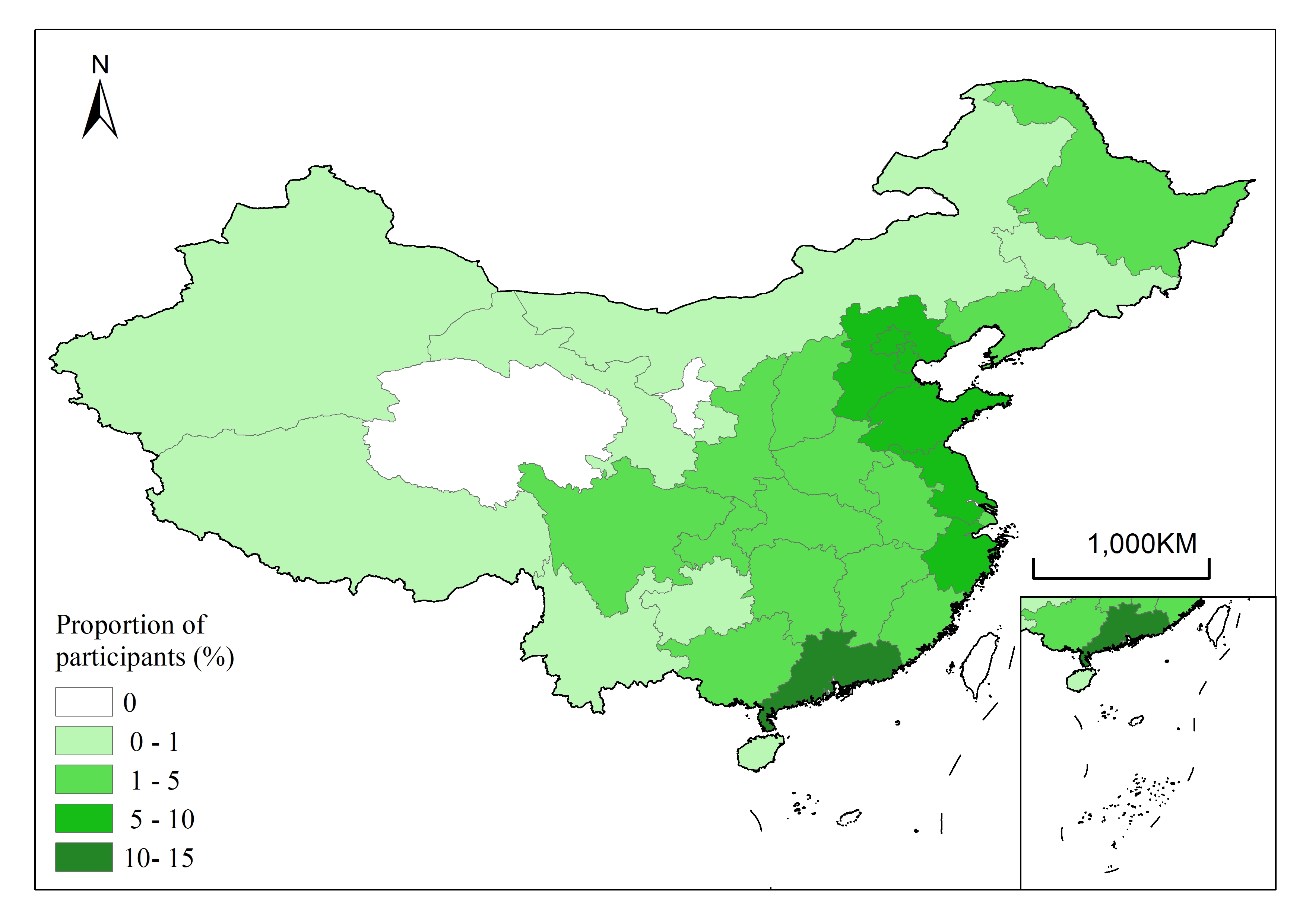


Appendix S6. Comparison of different questionnaire collection channels in pilot surveys.

| ***Collection channels*** | ***Duration of sampling*** | ***Total samples*** | ***Effective samples*** | ***Target people reach ratio*** |
| --- | --- | --- | --- | --- |
| Wenjuanxing (WJX) | 33 days | 485 | 300 | 61.86% |
| Baidu | 2 hours | 211 | 57 | 27.01% |
| Fangcun | 4 hours | 139 | 35 | 25.18% |
| University | 1 day | 254 | 26 | 10.24% |

Appendix S7. Table Model selection using AIC and BIC for the Random Parameter Logit (RPL) models.

| ***Model*** | ***Interaction*** | ***AIC*** | ***BIC*** |
| --- | --- | --- | --- |
| Model 1 | No interaction | 16759.83 | 16875.04 |
| Model 2 | Exposure level | 16767.41 | 16943.43 |
| Model 3 | Age | 16791.34 | 16956.95 |
| Model 4 | Gender | 16778.4 | 16944.02 |
| Model 5 | Education | 16797.16 | 16962.78 |
| Model 6 | Income | 16775.67 | 16941.29 |
| Model 7 | Number of turtles kept | 16789.82 | 16955.44 |
| Model 8 | Years of breeding experience | 16797.16 | 16962.78 |
| Model 9 | Social influence | 16756 | 16921.62 |
| Model 10 | The score of captive-bred definition knowledge | 16801.39 | 16967.01 |
| Model 11 | The score of freshwater turtle legality knowledge | 16787.13 | 16952.74 |
| Model 12 | Exposure level + Social influence | 16751.3 | 17017.73 |

Appendix S8. Summary of measures of model fit for the Latent Class Logit (LCL) Models. The model with the lowest AIC and BIC and at least 1% for the lowest class for each class choice is selected (i.e., Model 2) (Natali et al. 2022).

| ***Model*** |  | ***With covariate*** | ***AIC*** | ***BIC*** |
| --- | --- | --- | --- | --- |
| Model 1 |  | 2-Class Choice | 17120.67 | 17315.09 |
| Model 2 |  | 3-Class Choice | 16565.73 | 16889.77 |
| Model 3 |  | 4-Class Choice | 16589.49 | 17043.14 |
| Model 4 |  | 5-Class Choice | 16889.77 | 16891.09 |

Appendix S9. Summary of the Multinominal Logit (MNL) estimates from the discrete choice experiment on consumer preferences (N = 1981), including standard errors in parentheses. Significance levels: *P < 0.05, **P < 0.01.

| ***Attribute*** | ***Level*** | **Estimate (sd)** |  |
| --- | --- | --- | --- |
| Population trends in the wild  *(Ref. Decreasing)* | Increasing | 0.32**  (0.06) | |
|  | Stable | 0.19**  (0.05) | |
| Captive breeding techniques  *(Ref. Early development)* | Well-developed | 0.08*  (0.04) | |
| Method of purchase  *(Ref. Online, delivery by mail)* | Offline, collection in-person | -0.45**  (0.03) | |
| Relevant seller certificates  *(Ref. Not displayed)* | Displayed | 0.59**  (0.03) | |
| Individual source  *(Ref. Wild-caught)* | Captive-bred | 0.53**  (0.03) | |
| Price (CNY /individual) |  | -0.50**  (0.03) | |
| ASC |  | -1.51**  (0.02) | |
| **AIC** | 18940.29 |  | |
| **BIC** | 19005.1 |  | |
| **Log-Likelihood** | -9461.14 |  | |
| **Number of individuals** | 1981 |  | |
| **Number of rows** | 9905 |  | |

Appendix S10 Questionnaire

**Pet turtle keeping experience and choice experiment survey**

**宠物龟饲养经历及认知调查**

This survey is part of a project that looks at the **experiences with keeping turtles** **and pet consumption habits of Chinese nationals residing in Guangdong province, China**. This research is part of a wider project administered by <REDACTED>, co-managed and funded by the <REDACTED>.

The survey will take approximately **10-15** minutes to complete. Respondents who fully complete the survey may receive member reward points for participation.

As this is an anonymous survey, your answers will be processed in code, thus will not be asked for your name or other identifying information for privacy and security reasons. This survey may include questions that could be perceived as sensitive, but your participation will have no way of being traced back to you. If you encounter any issues during the survey, you can withdraw at any time without reason or negative consequences. All findings from this study are limited to usage for academic research only.

This project has been reviewed for research ethics by the <REDACTED> (reference code #22-37). If you have any concern about any aspect of this project, please contact <REDACTED>.

此问卷是调查公众宠物龟饲养经历及认知研究项目的一部分。该项目由<REDACTED>及其合作伙伴<REDACTED>共同出资并管理。

感谢您抽时间参与我们问卷调查，填写此问卷大约需要10分钟。

出于隐私和安全考虑，此调查完全匿名且无需提供任何个人身份信息。此问卷可能会涉及一些敏感问题，但您的回答无法追溯回您本人，且问卷无法识别出受访者，您的答案也将会转换为代码后处理。在填写过程中您若有疑问可以随时无理由退出，且不会有任何不良后果。此项研究的所有结果将仅限用于学术研究。

此项目已通过<REDACTED>伦理委员会的审查，编号为#22-37 。若您对项目的任何方面有疑问，欢迎邮件联系<REDACTED>

*[consent]* **Do you agree to take part in this survey? 您是否同意参与此次调查？**

- Yes 是

- No 否

1. What is your age group: [Single Choice]

您的年龄段：[单选题]

○Under 18 years 18岁以下; ○18-24 years 18岁 - 24岁;

○25-29 years 25岁 - 29岁; ○30-34 years 30岁 - 34岁;

○35-39 years 35岁 - 39岁; ○40-44 years 40岁 - 44岁;

○45-49 years 45岁 - 49岁; ○50-54 years 50岁 - 54岁;

○55 years and above 55岁以上

2. To date, how many individual pet turtles have you ever kept in total? [Single Choice] 您从开始养龟到目前为止**一共**饲养过多少只宠物龟 [单选题]

○0 individuals 0只○1 individual 1只

○2-5 individuals 2 - 5 只○6-10 individuals 6 - 10 只

○11-20 individuals 11 - 20 只○Over 20 individuals 20只及以上

3.What channels/platforms do you generally follow[/use] to seek relevant content about pet turtles? [Multiple Choice]

您平时关注**宠物龟相关**内容的途径有哪些？ [多选题]

**Online 线上: Video-sharing platforms 视频平台**

□ Tik tok 抖音□ Kuaishou 快手□ Bilibili 哔哩哔哩

**Online: Q&A enquiry sites 问答网站**

□ Baidu Zhidao 百度知道

□ Zhihu 知乎

**Online: Other platforms 其他线上平台**

□Official accounts (please specify)__________ 百度贴吧 请注明至少一个常去的贴吧名

□ Discussion forums (please specify)__________ 公众号 请注明至少一个常去的公众号

□ E-commerce platforms (please specify),__________ 论坛 请注明至少一个常去的论坛名

□ (Social media) Groupchats, e.g. QQ, WeChat QQ或微信等线上群聊

**Offline platforms 线下**

□ Turtle (hobbyist) shows 龟展

□ Zoos, aquariums 动物园、水族馆

**Other其他**

□ Other,___________________ 其他（请注明）

4. In total, how long have you ever kept pet turtles for, to date? [Single Choice]

您目前一共饲养宠物龟多久了 [单选题]

It refers to a total of pet turtles from the beginning to the present

指从最初养宠物龟到现在为止一共

○ Less than 1 year 不到1年

○ 1-5 years 1-2年

○ 6-10 years 3-5年

○ Over 10 years 6-10年

5. Have you ever hear of the following species of turtles before? [Matrix single-choice] *

您是否**听说过**以下龟种？[矩阵单选题]

|  | Have heard of  听说过 | Never heard of  没听说过 | Not sure  我不确定 |
| --- | --- | --- | --- |
| Indochinese box turtle (*Cuora galbinifrons / Cuora bourreti / Cuora picturata*)  黄额闭壳龟（包括黑腹、图画、布氏） | ○ | ○ | ○ |
| Keeled box turtle (*Cuora mouhotii*)  锯缘闭壳龟（锯缘摄龟） | ○ | ○ | ○ |
| Southeast Asian box turtle (*Cuora amboinensis*)  马来闭壳龟（安布闭壳龟） | ○ | ○ | ○ |
| Big-headed turtle (*Platysternon megacephalum*)  鹰嘴龟（平胸龟） | ○ | ○ | ○ |
| Snapping turtle (incl. *Macroclemys temminckii / Chelydra serpentina*)  鳄龟（包括大鳄龟、小鳄龟） | ○ | ○ | ○ |
| Stink-pot turtle  麝香龟 | ○ | ○ | ○ |
| Red-eared slider  巴西龟（红耳巴西龟） | ○ | ○ | ○ |

6. Have you ever kept the following turtle species as pets? [Matrix single-choice]

您是否**饲养过**以下龟种？[矩阵单选题]

|  | Have kept before  曾经养过 | Currently keeping  正在养 | Never kept before  没养过 | Not sure  我不确定 | |
| --- | --- | --- | --- | --- | --- |
| Indochinese box turtle (*Cuora galbinifrons / Cuora bourreti / Cuora picturata*)  黄额闭壳龟 | ○ | ○ | ○ | ○ |  |
| Keeled box turtle (*Cuora mouhotii*)  锯缘闭壳龟 | ○ | ○ | ○ | ○ |  |
| Southeast Asian box turtle (*Cuora amboinensis*)  马来闭壳龟 | ○ | ○ | ○ | ○ |  |
| Big-headed turtle (*Platysternon megacephalum*)  鹰嘴龟 | ○ | ○ | ○ | ○ |  |
| Snapping turtle (incl. *Macroclemys temminckii / Chelydra serpentina*)  鳄龟 | ○ | ○ | ○ | ○ |  |
| Stink-pot turtle  麝香龟 | ○ | ○ | ○ | ○ |  |
| Red-eared slider  巴西龟 | ○ | ○ | ○ | ○ |  |
| Other turtle species  其它龟种 | ○ | ○ | ○ | ○ |  |

7. In addition to the specific turtle species that appeared in the previous question, what other turtle species have you ever kept? [Fill in the blank]

除上一题出现过的具体龟种，您还饲养过哪些龟种？ [填空题] *

_________________________________

Rely on option 1&2 in the heading of the 8th row of question 6

依赖于第6题第8个行标题中的第1;2个选项

8. Please select the Big-headed turtle among the following pictures within the specified time [Single Choice]

请在**规定时间内**选出下列中的**平胸龟（鹰嘴龟）**[单选题]

**
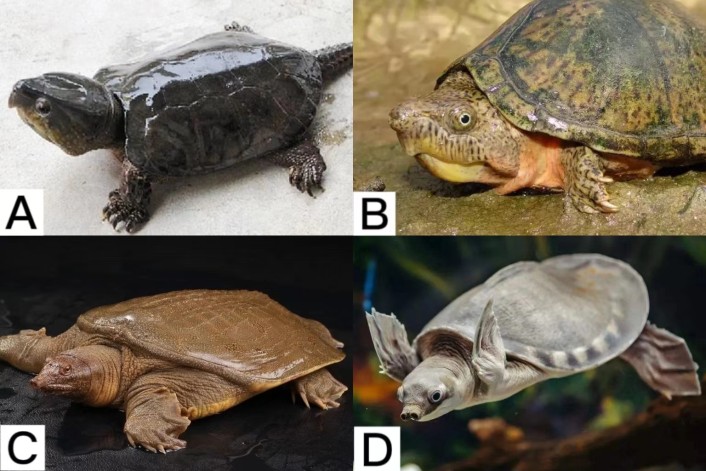
**

| ○A ○B ○C ○D |
| --- |

Rely on option 1&2 in the heading of the 4th row of question 6

依赖于第6题第4个行标题中的第1;2个选项

9. Please select the Indochinese box turtle among the following pictures within the specified time [Single Choice]

请在**规定时间内**选出下列中的**黄额闭壳龟（黄额盒龟）**[单选题]

**
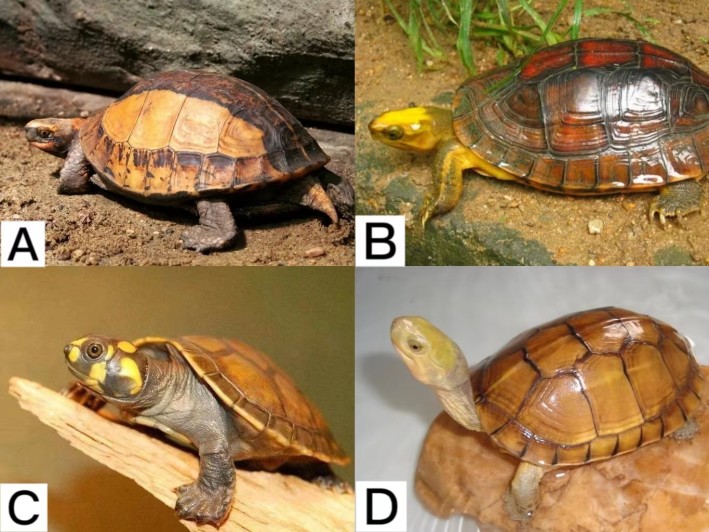
**

| ○A ○B ○C ○D |
| --- |

Rely on option 1&2 in the heading of the 1st row of question 6

依赖于第6题第1个行标题中的第1;2个选项

10. Please select the Kneeled box turtle among the following pictures within the specified time [Single Choice]

请在**规定时间内**选出下列中的**锯缘摄龟（锯缘闭壳龟））**[单选题]


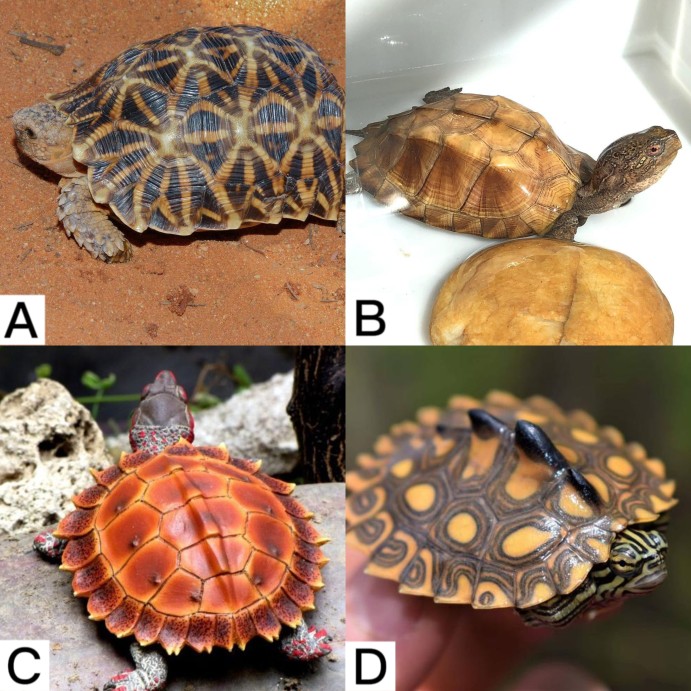


| ○A ○B ○C ○D |
| --- |

Rely on option 1&2 in the heading of the 2nd row of question 6

依赖于第6题第1个行标题中的第1;2个选项

11. Please select the Southeast Asian box turtle among the following pictures within the specified time [Single Choice]

请在**规定时间内**选出下列中的**马来闭壳龟（安布闭壳龟）**[单选题]

**
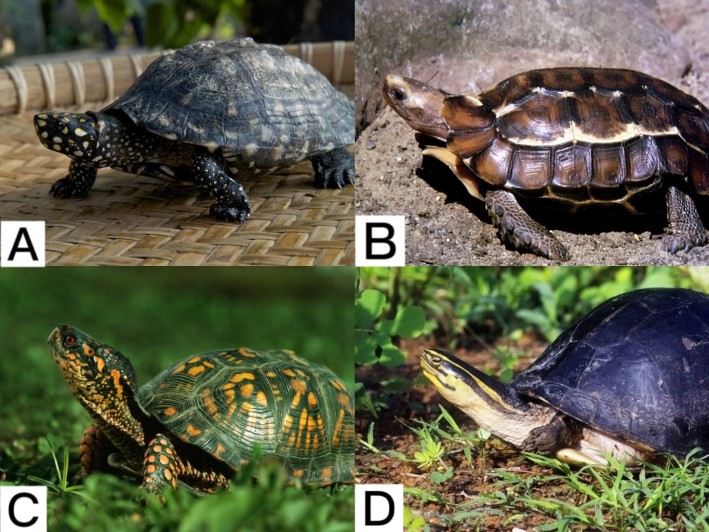
**

○A ○B ○C ○D

Rely on option 1&2 in the heading of the 3rd row of question 6

依赖于第6题第3个行标题中的第1;2个选项

12. Have you heard of other people from your social circle who has or had ever kept the following turtle species as pets? [Matrix multiple-choice]

您是否听说过社交圈内的人**饲养过**以下龟种？[矩阵多选题]

|  | Family  家人 | Friends  朋友 | Classmates/ colleagues  同学或同事 | As far as I know, no  据我所知没有 |
| --- | --- | --- | --- | --- |
| Indochinese box turtle (*Cuora galbinifrons / Cuora bourreti / Cuora picturata*)  黄额闭壳龟 | □ | □ | □ | □ |
| Keeled box turtle (*Cuora mouhotii*)  锯缘闭壳龟 | □ | □ | □ | □ |
| Southeast Asian box turtle (Cuora amboinensis)  马来闭壳龟 | □ | □ | □ | □ |
| Big-headed turtle (*Platysternon megacephalum*)  鹰嘴龟 | □ | □ | □ | □ |
| Snapping turtle (incl. *Macroclemys temminckii / Chelydra serpentina*)  鳄龟 | □ | □ | □ | □ |
| Stink-pot turtle  麝香龟 | □ | □ | □ | □ |
| Red-eared slider  巴西龟 | □ | □ | □ | □ |
| Other turtle species  其他龟 | □ | □ | □ | □ |

13. What are the main reasons for your choice to keep turtles as pets? [Multiple Choice]

您选择龟作为宠物的主要原因是？ [多选题]

| □Easy to keep or raise and take care of or maintain容易饲养和打理 |
| --- |
| □Companionship陪伴 |
| □Novelty, special新奇、特别 |
| □For financial investment投资 |
| □Wish to attempt with breeding or cross-breeding turtles想要尝试龟的繁殖或杂交 |
| □Others _________________其他 |
| □No particular reason没有特别的原因 |
| □Not sure我不确定 |

14. What are the reasons for your choice to keep *Cuora spp.* box turtles as pets? [Multiple Choice]

在宠物龟中，您选择闭壳龟这一龟种的原因是 [多选题]

**Physical appearance 外表**

□ Exhibits features that match my aesthetic interests

长相符合我的审美

**Care level/husbandry difficulty 饲养难度**

□ Low in difficulty to me, easy to keep alive 对我来说饲养难度低，好养活

□ High in difficulty to me, up for the challenge 对我来说饲养难度高，有挑战性

**Personality [trait] 性格**

□ Docile and calm 性格温顺

□ Aggressive and dominant 性格凶猛

I**nteractivity 互动性**

□ Enjoys human interaction 喜欢和人互动

□ Does not require constant interaction 不需要经常互动

**Other 其他**

□ Visual enjoyment or appreciation of particular characteristic behaviors or acts 某些行为具有观赏性

□ Culturally symbolic 具有文化象征

□ Uncommon in the market 在市面上不常见

□ Other, ____ 其他

□ No particular reason 没有特别的原因

Rely on 1&2 options in the header of the first, second and third row of question 6

依赖于第6题第1个行标题中的第1;2个选项，第6题第2个行标题中的第1;2个选项，第6题第3个行标题中的第1;2个选项

15. What are the reasons for your choice to keep Big-headed turtle as pets? [Multiple Choice]

在宠物龟中，您选择鹰嘴龟这一龟种的原因是 [多选题]

**Physical appearance 外表**

□ Exhibits features that match my aesthetic interests

长相符合我的审美

**Care level/husbandry difficulty 饲养难度**

□ Low in difficulty to me, easy to keep alive 对我来说饲养难度低，好养活

□ High in difficulty to me, up for the challenge 对我来说饲养难度高，有挑战性

**Personality [trait] 性格**

□ Docile and calm 性格温顺

□ Aggressive and dominant 性格凶猛

I**nteractivity 互动性**

□ Enjoys human interaction 喜欢和人互动

□ Does not require constant interaction 不需要经常互动

**Other 其他**

□ Visual enjoyment or appreciation of particular characteristic behaviors or acts 某些行为具有观赏性

□ Culturally symbolic 具有文化象征

□ Uncommon in the market 在市面上不常见

□ Other, ____ 其他

Rely on 1&2 options in the header of the fourth row of question 6

依赖于第6题第4个行标题中的第1;2个选项

16. Which of the following ways have you ever used to acquire/obtain pet turtles previously? [Multiple Choice]

您通过以下哪些途径获得过宠物龟 [多选题]

| □Purchased by myself自己购入 |
| --- |
| □Gifted by others他人赠与 |
| □Adopted or rescued领养或救治 |
| □Acquired or found outdoors or from the wild野外获取或捡来的 |
| □Others_________________其他 |

17. Where did you or What channels have you ever used to purchase pet turtle(s) from? [Multiple Choice]

您购入宠物龟的途径是？ [多选题]

| □Selections from posts on the Traders' WeChat Moments商家的微信朋友圈 |
| --- |
| □Xianyu, Taobao and/or other e-commerce websites咸鱼、淘宝等购物网站 |
| □Turtle hobbyist shows龟展 |
| □Commercial or breeding farms养殖户 |
| □Fish, flower and bird markets花鸟鱼虫市场 |
| □Reptile pet stores爬虫线下专卖店 |
| □Secondhand from other turtle hobbyists龟友二手转卖 |
| □Other_________________其他 |

Rely on option 1 of question 16

依赖于第16题第1个选项

18. From whom had you acquired/obtained your pet turtle(s) from previously? [Multiple Choice]

您是从谁那里获得您的宠物龟的 [多选题]

| □Family or friends亲友 |
| --- |
| □Classmates or colleagues同学或同事 |
| □Other turtle hobbyists龟友 |
| □Other_________________其他 |
| □Prefer not to say不愿透露 |

Rely on option 1&2 of question 16

依赖于第16题第2;3个选项

19. What factors do you tend to consider or emphasize on the most when choosing between turtle sellers? [Multiple Choice]

您在选择**宠物龟卖方**时主要会考虑哪些因素？ [多选题]

| □A valid merchant license具有营业资格 |
| --- |
| □Offers me with legal petkeeping certificates能够为我提供合法养龟证件 |
| □Services that are able to help me get legal petkeeping certificates能够代办养殖需要的证件 |
| □Recommendations by close acquaintances熟人推荐 |
| □Positive reviews有良好的口碑 |
| □Diverse supplies or selections of turtle species龟种繁多 |
| □Offers great value-for-money with sales of turtle species卖的龟种性价比高 |
| □Other _________________其他 |
| □No particular reason or consideration, chosen randomly没有特别的考虑，随便挑选 |

Rely on option 1 of question 16

依赖于第16题第1个选项

20. Which of the following turtles do you believe are considered “captive-bred” individuals? [Multiple Choice]

您认为以下哪些龟为**人工繁育**个体？


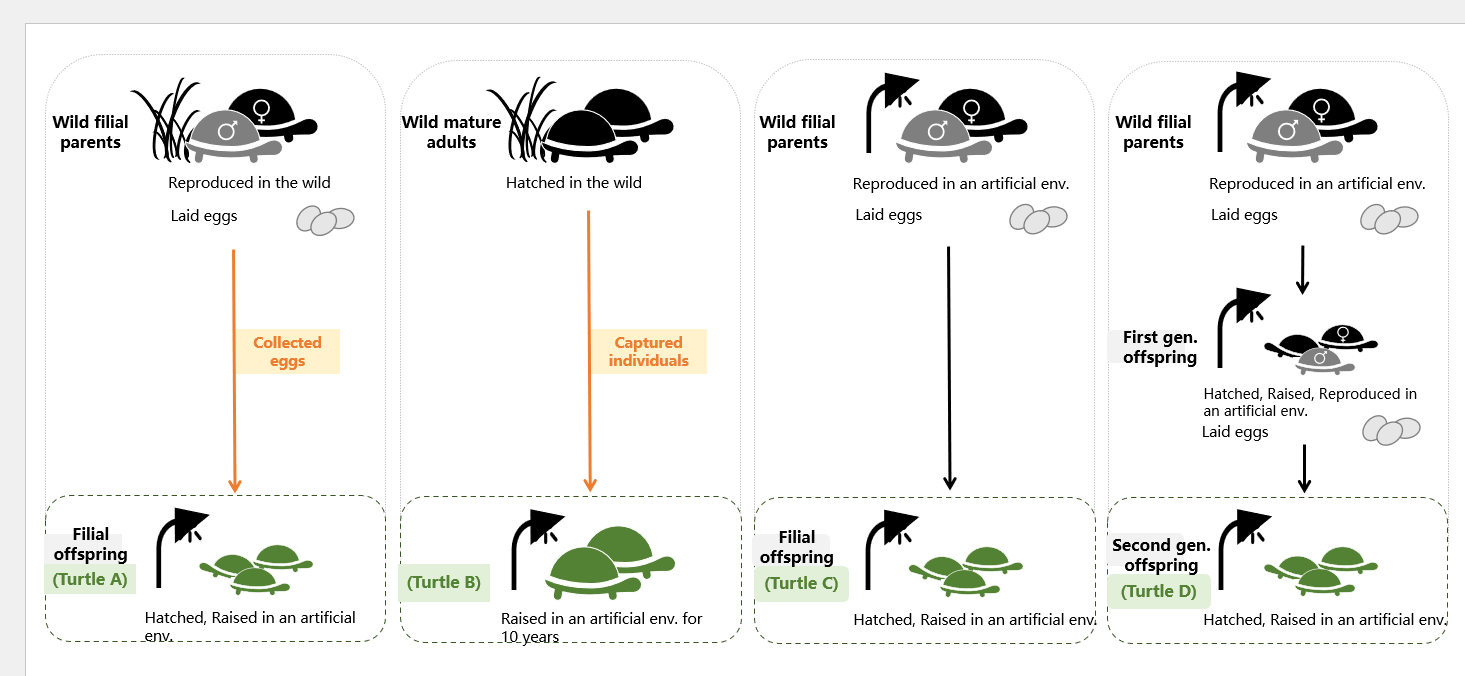


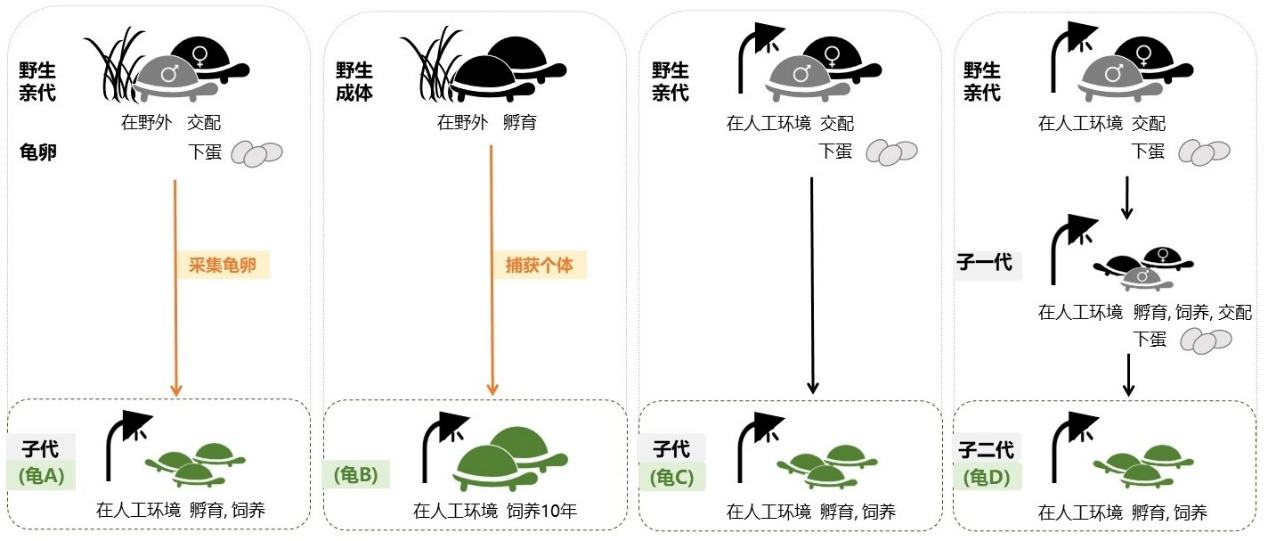


| □Turtle A龟A  Eggs collected from the wild and hatched Turtle A in an artificial env.  从野外采集龟卵，在人工环境下孵化为龟A |
| --- |
| □Turtle B龟B  Individuals captured from the wild and raised in an artificial env. for 10 years  从野外捕获个体，人工环境饲养十年 |
| □Turtle C龟C  Wild filial parents reproduce and hatched Turtle C in an artificial env.  野外亲代在人工环境下繁殖、孵化出龟C |
| □Turtle D龟D  Wild filial parents reproduce and hatched offspring in an artificial env.; then the offspring reproduce and hatched Turtle D in an artificial env.  野外亲代在人工环境下繁殖、孵化出子代，子代在人工环境下繁殖、孵育出龟D |
| □None of the above |

都不是

21. Do you have any plans or intentions to purchase of the following species of turtles in the near future? [Multiple Choice]

您未来是否有购入以下龟作为宠物的打算？ [多选题]

| □ | Indochinese box turtle (*Cuora galbinifrons / Cuora bourreti / Cuora picturata*) 黄额闭壳龟 |
| --- | --- |
| □ | Keeled box turtle (*Cuora mouhotii*) 锯缘闭壳龟 |
| □ | Southeast Asian box turtle (*Cuora amboinensis*) 马来闭壳龟 |
| □ | Big-headed turtle (*Platysternon megacephalum*) 鹰嘴龟 |
| □ | Snapping turtle (incl. *Macroclemys temminckii / Chelydra serpentina*) 鳄龟 |
| □ | Stink-pot turtle麝香龟 |
| □ | Red-eared slider巴西龟 |
| □ | Other species_________________其他龟种 |
| □ | Have plans or intend to purchase turtles, but have not decided on a particular species yet有买龟的打算，但是还未确定龟种 |
| □ | Have no plans or intentions to purchase turtles at the moment目前没有购买龟的打算 |

22. Attention Check, please select “Somewhat disagree” [Single choice]

本题为注意力测试题，请您选择 “比较不同意” 这一选项 [单选题]

| ○Strongly agree非常同意 |
| --- |
| ○Somewhat agree比较同意 |
| ○Somewhat disagree比较不同意 |
| ○Strongly disagree非常不同意 |

**Consumption preference: survey instruction**

**消费偏好: 问卷调查指南**

23.

(1) The following six questions describe a hypothetical buying scenario. Please read the following information we have provided before proceeding (including blue text on turtle species conditions outlined within the question, and listed turtle attributes in the tables included below). Based on the given details, you can choose to either purchase one of two types of turtles, or to purchase neither option.

针对以下5个情景问题，请您先阅读我们所给的信息（包括题干中蓝色字体标注的龟种现状，以及图表下的龟种属性）。根据这些信息，您可以选择购买三个龟种之一或三种都不买。

(2) Questions in this section will be displayed in the format illustrated in the image below. Please read the following content carefully to ensure that you fully understand the information given, and are aware of how to interpret these questions before proceeding to respond.
调查问题将以下列图片的格式显示，现请阅读以下内容，确保您在开始回答问题前已知晓我们所给的信息以及阅读方法。您的回答都将是匿名的，答案无法回溯到您本人。

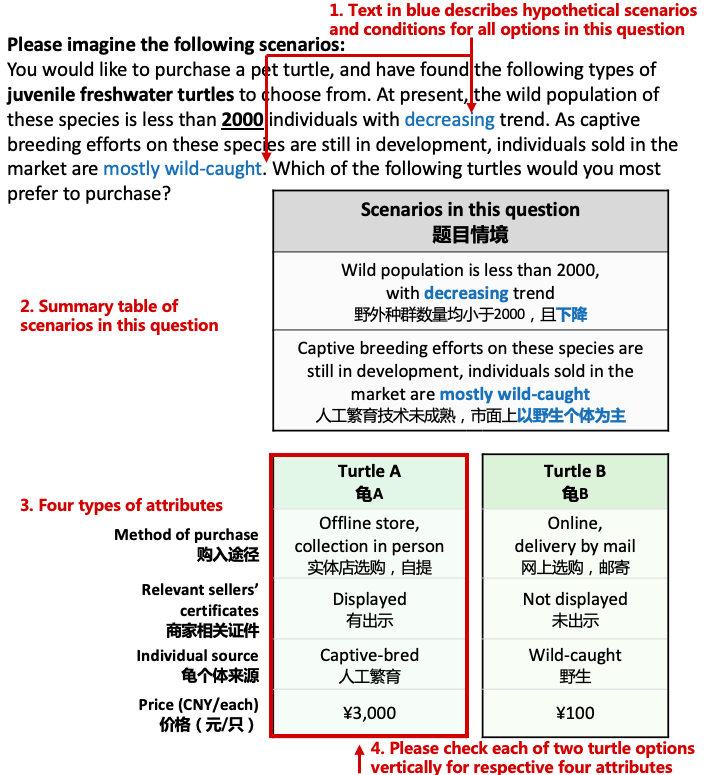


(3) As your answers are anonymized, your responses are unable to be traced back to you personally. 您的回答都将是匿名的，答案无法回溯到您本人。

Please select “I understand” to indicate that you have fully read and understood the instructions stated. [Single choice]

请勾选“我已知晓”，以表示您对所提供的说明信息已完全知晓并理解。 [单选题]

| ○ I understand我已知晓 |
| --- |

24. **Please select “I understand” to indicate that you have read and fully understood [all] the following statements and information provided.**

**请勾选“我已知晓”，以表示您对所提供的说明信息已完全知晓并理解**

○I understand that “**juvenile freshwater turtles**,” as noted within the question, refer only to individuals with an average shell length of 3-5cm.

我已知晓，题目中 “龟苗” 指背壳长度约为3-5cm的幼龟

○I understand that “**relevant sellers’ certificates**” refers to any potential licensing or legal documentation that is required of the seller, for commercial business, breeding and/or sourcing of turtle species, etc.

□我已知晓，商家的 “相关证件” 包括经营、繁育、龟种来源等所有商家可能所需的证件

○I understand that “**wild-caught individuals**” refer to turtles that are hatched from eggs laid in the wild.

我已知晓， “野生个体” 指从野外捕获的龟卵中孵化出的龟个体

○I understand that “**captive-bred individuals**” refer to turtles that are filial offspring of parents born, raised and reproduced in an artificial environment or captivity.

我已知晓， “人工繁育个体” 指亲代在人工环境下生长、交配、繁育的子后代

25. The following few questions have differences in scenarios and options, please read it carefully. You may click the photo to take a close look. [Single choice]

以下几题在情景与选项上有所不同，请您仔细阅读。

**Respondents would assigned randomly to one of the three subsets of Choice experiment.**

**受访者将被随机分入不同的组**

**Subsets 1: Q26-Q31**

**第一组：26-31**

26. **Please imagine the following scenarios:** 请您假设以下情景：

You would like to purchase a pet turtle, and have found the following types of **juvenile freshwater turtles** to choose from. At present, the wild population of these species is less than **2000** individuals with decreasing trend. Captive breeding efforts on these species are developed, and abundant captive-bred individuals are sold in the market. Which of the following turtles would you most prefer to purchase? [Single choice]

您想要选购一只宠物龟，现有下列几种淡水龟龟苗供您选择。它们的野外种群数量均小于2000，数量仍在下降，但人工繁育技术成熟，市面上以有大量人工繁育个体。您更倾向于购买下列哪一只龟？[单选题]


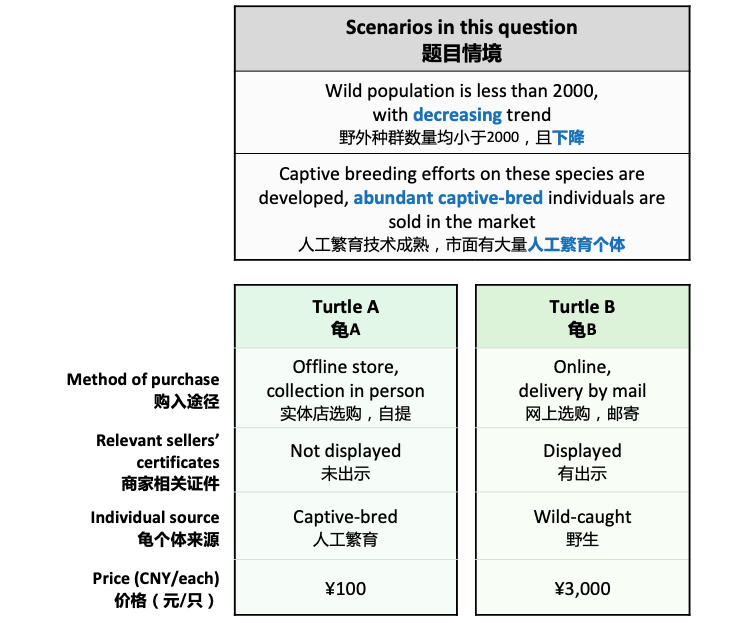


| ○Turtle A龟种A |
| --- |
| ○Turtle B龟种B |
| ○Neither of the turtles above would I like to purchase以上龟种我都不想购买 |

27. **Please imagine the following scenarios:** 请您假设以下情景

You would like to purchase a pet turtle, and have found the following types of **juvenile freshwater turtles** to choose from. At present, the wild population of these species is less than **2000** individuals with decreasing trend. Captive breeding efforts on these species are developed, and abundant captive-bred individuals are sold in the market. Which of the following turtles would you most prefer to purchase? [Single choice]

您想要选购一只宠物龟，现有下列几种淡水龟龟苗供您选择。它们的野外种群数量均小于2000，数量仍在下降，但人工繁育技术成熟，目前市面上有大量人工繁育个体。您更倾向于购买下列哪一只龟？[单选题]


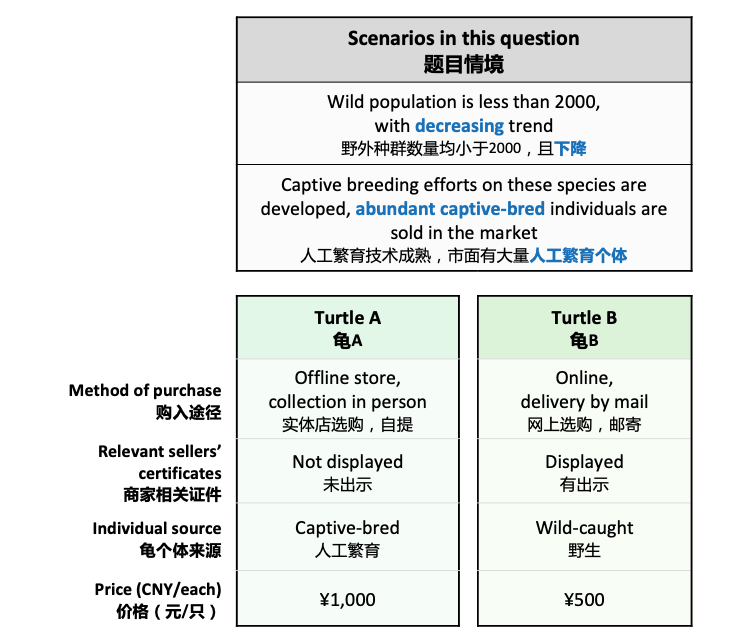


| ○Turtle A龟种A |
| --- |
| ○Turtle B龟种B |
| ○Neither of the turtles above would I like to purchase以上龟种我都不想购买 |

28. **Please imagine the following scenarios:** 请您假设以下情景

You would like to purchase a pet turtle, and have found the following types of **juvenile freshwater turtles** to choose from. At present, the wild population of these species is less than **2000** individuals with stable population. As captive breeding efforts on these species are still in development, individuals sold in the market are mostly wild-caught. Which of the following turtles would you most prefer to purchase? [Single choice] *您想要选购一只宠物龟，现有下列几种淡水龟龟苗供您选择。它们的野外种群数量均小于2000，数量稳定，但人工繁育技术未成熟，市面上以野生个体为主。您更倾向于购买下列哪一只龟？[单选题]


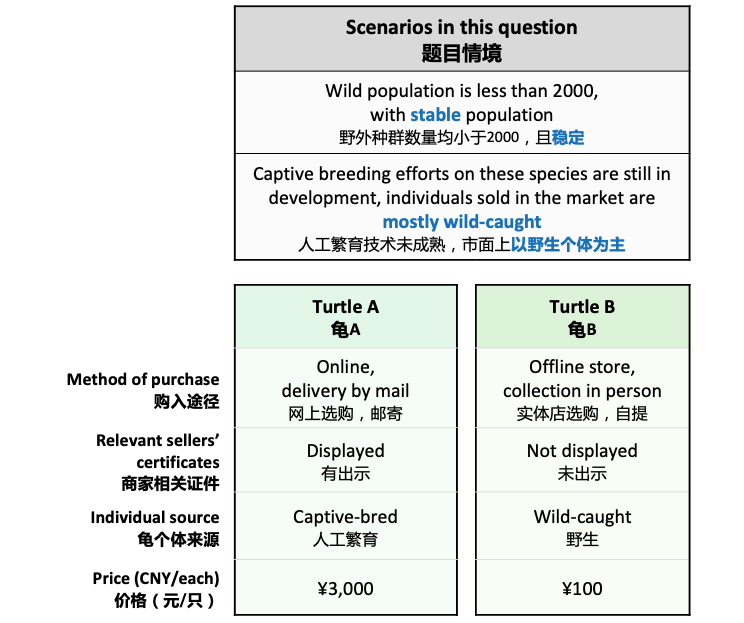


| ○Turtle A龟种A |
| --- |
| ○Turtle B龟种B |
| ○Neither of the turtles above would I like to purchase以上龟种我都不想购买 |

29. **Please imagine the following scenarios:** 请您假设以下情景

You would like to purchase a pet turtle, and have found the following types of **juvenile freshwater turtles** to choose from. At present, the wild population of these species is less than **2000** individuals with stable population. As captive breeding efforts on these species are still in development, individuals sold in the market are mostly wild-caught. Which of the following turtles would you most prefer to purchase? [Single choice]

您想要选购一只宠物龟，现有下列几种淡水龟龟种供您选择。它们的野外种群数量均小于2000，数量稳定，但人工繁育技术未成熟，市面上以野生个体为主。您更倾向于购买下列哪一只龟？[单选题]


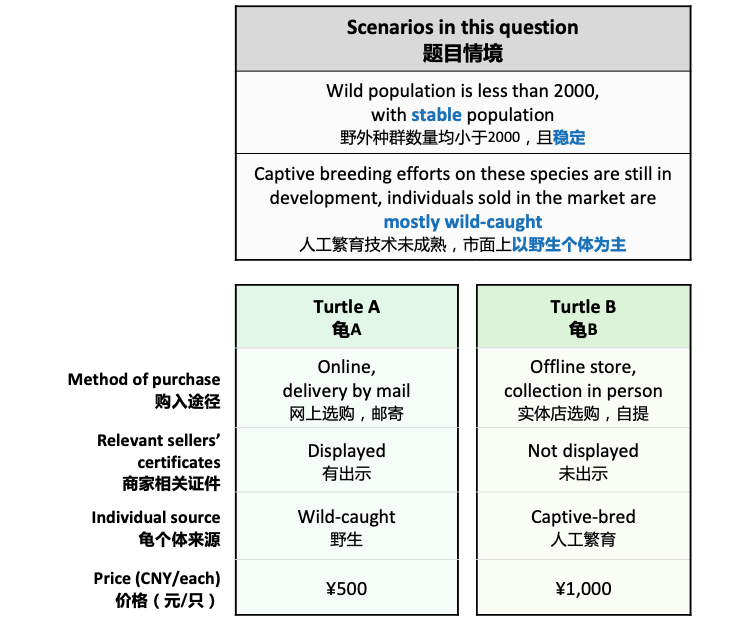


| ○Turtle A龟种A |
| --- |
| ○Turtle B龟种B |
| ○Neither of the turtles above would I like to purchase以上龟种我都不想购买 |

30. **Please imagine the following scenarios:** 请您假设以下情景：

You would like to purchase a pet turtle, and have found the following types of **juvenile freshwater turtles** to choose from. At present, the wild population of these species is less than **2000** individuals with increasing trend. Captive breeding efforts on these species are developed, and abundant captive-bred individuals are sold in the market. Which of the following turtles would you most prefer to purchase? [Single choice]

您想要选购一只宠物龟，现有下列几种**淡水龟龟苗**供您选择。它们的野外种群数量均小于**2000**，数量正在**上升**，且人工繁育技术成熟，目前市面上有**大量人工繁育个体**。您更倾向于购买下列哪一只龟？[单选题]


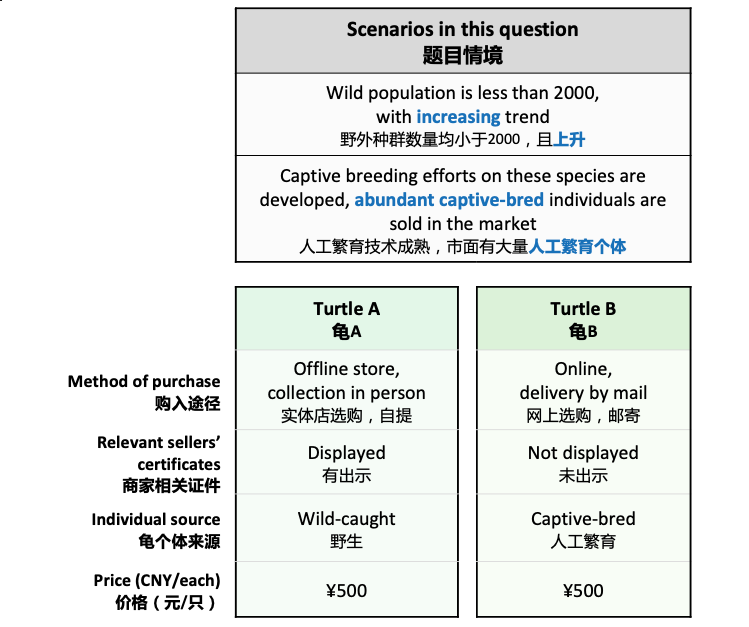


| ○Turtle A龟种A |
| --- |
| ○Turtle B龟种B |
| ○Neither of the turtles above would I like to purchase以上龟种我都不想购买 |

31. **Please imagine the following scenarios:** 请您假设以下情景：

You would like to purchase a pet turtle, and have found the following types of **juvenile freshwater turtles** to choose from. At present, the wild population of these species is less than **2000** individuals with decreasing trend. Captive breeding efforts on these species are developed, and abundant captive-bred individuals are sold in the market. Which of the following turtles would you most prefer to purchase? [Single choice]

您想要选购一只宠物龟，现有下列几种**淡水龟龟苗**供您选择。它们的野外种群数量均小于**2000**，数量仍在**下降**，但人工繁育技术成熟，市面上有**大量人工繁育个体**。您更倾向于购买下列哪一只龟？[单选题]


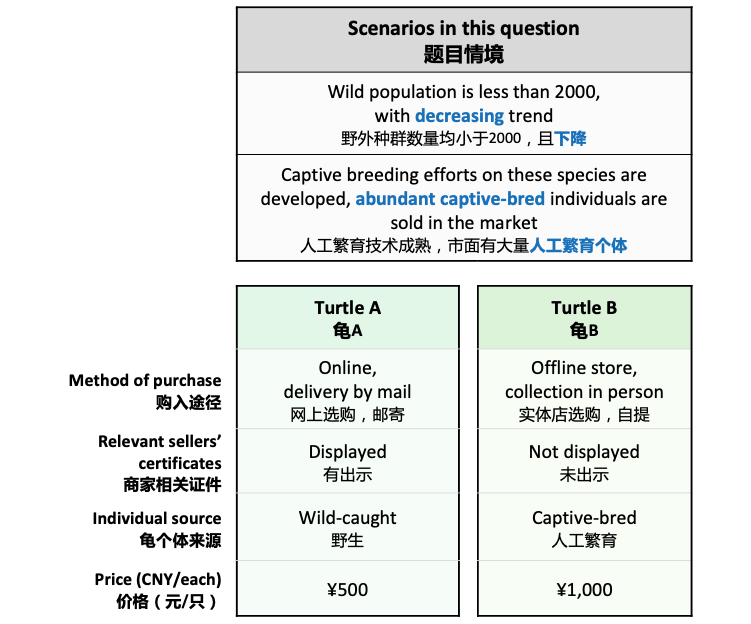


| ○Turtle A龟种A |
| --- |
| ○Turtle B龟种B |
| ○Neither of the turtles above would I like to purchase以上龟种我都不想购买 |

**Subsets 2: Q32-Q37**

**第一组：32-37**

32. **Please imagine the following scenarios:** 请您假设以下情景

You would like to purchase a pet turtle, and have found the following types of **juvenile freshwater turtles** to choose from. At present, the wild population of these species is less than **2000** individuals with decreasing trend. As captive breeding efforts on these species are still in development, individuals sold in the market are mostly wild-caught. Which of the following turtles would you most prefer to purchase? [Single choice]

您想要选购一只宠物龟，现有下列几种淡水龟龟苗供您选择。它们的野外种群数量均小于2000，数量仍在下降，且人工繁育技术未成熟，目前市面上以野生个体为主。您更倾向于购买下列哪一只龟？[单选题]


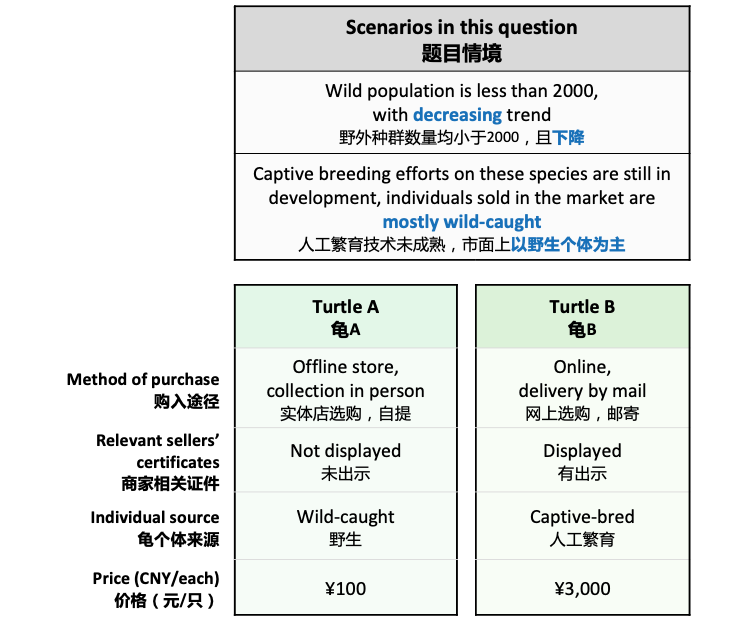


| ○Turtle A龟种A |
| --- |
| ○Turtle B龟种B |
| ○Neither of the turtles above would I like to purchase以上龟种我都不想购买 |

33. **Please imagine the following scenarios:** 请您假设以下情景

You would like to purchase a pet turtle, and have found the following types of **juvenile freshwater turtles** to choose from. At present, the wild population of these species is less than **2000** individuals with stable population. Captive breeding efforts on these species are developed, and abundant captive-bred individuals are sold in the market. Which of the following turtles would you most prefer to purchase? [Single choice]

您想要选购一只宠物龟，现有下列几种**淡水龟龟苗**供您选择。它们的野外种群数量均小于**2000**，数量**稳定**，且人工繁育技术成熟，目前市面上有**大量人工繁育个体**。您更倾向于购买下列哪一只龟？[单选题]


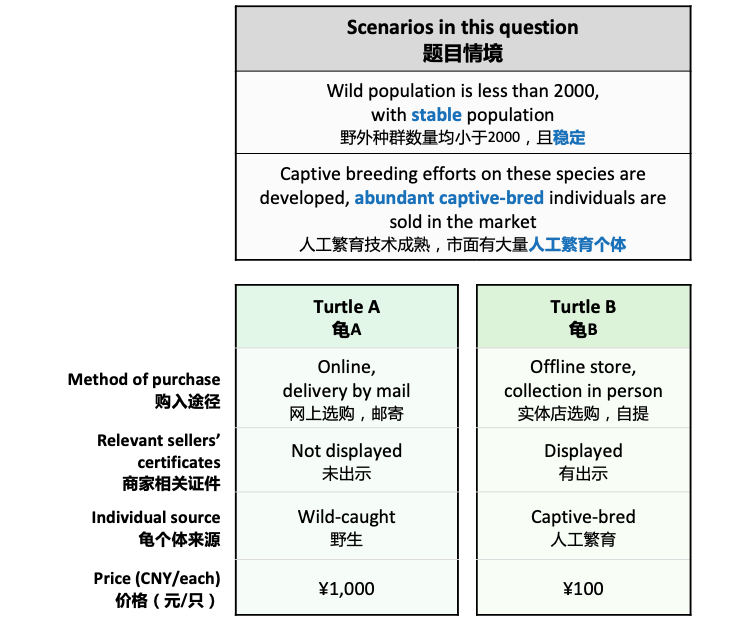


| ○Turtle A龟种A |
| --- |
| ○Turtle B龟种B |
| ○Neither of the turtles above would I like to purchase以上龟种我都不想购买 |

34. **Please imagine the following scenarios:** 请您假设以下情景

You would like to purchase a pet turtle, and have found the following types of **juvenile freshwater turtles** to choose from. At present, the wild population of these species is less than **2000** individuals with increasing trend. As captive breeding efforts on these species are still in development, individuals sold in the market are mostly wild-caught. Which of the following turtles would you most prefer to purchase? [Single choice]

您想要选购一只宠物龟，现有下列几种**淡水龟龟苗**供您选择。它们的野外种群数量均小于**2000**，数量正在**上升**，但人工繁育技术未成熟，市面上以**野生个体为主**。您更倾向于购买下列哪一只龟？[单选题]


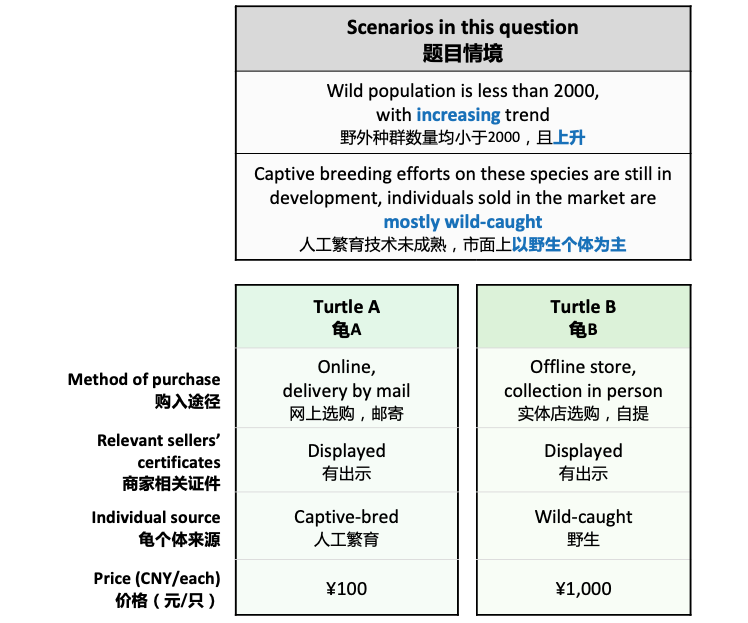


| ○Turtle A龟种A |  |
| --- | --- |
| ○Turtle B龟种B |  |
| ○Neither of the turtles above would I like to purchase以上龟种我都不想购买 |  |

35. **Please imagine the following scenarios:** 请您假设以下情景：

You would like to purchase a pet turtle, and have found the following types of **juvenile freshwater turtles** to choose from. At present, the wild population of these species is less than **2000** individuals with increasing trend. Captive breeding efforts on these species are developed, and abundant captive-bred individuals are sold in the market. Which of the following turtles would you most prefer to purchase? [Single choice]

您想要选购一只宠物龟，现有下列几种**淡水龟龟苗**供您选择。它们的野外种群数量均小于**2000**，数量正在**上升**，且人工繁育技术成熟，市面上有**大量人工繁育个体**。您更倾向于购买下列哪一只龟？[单选题]


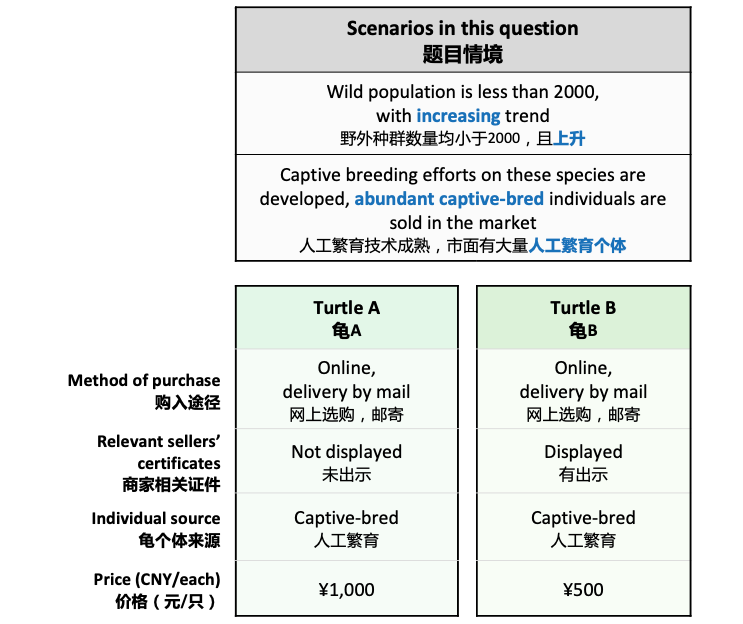


| ○Turtle A龟种A |
| --- |
| ○Turtle B龟种B |
| ○Neither of the turtles above would I like to purchase以上龟种我都不想购买 |

36. **Please imagine the following scenarios:** 请您假设以下情景：

You would like to purchase a pet turtle, and have found the following types of **juvenile freshwater turtles** to choose from. At present, the wild population of these species is less than **2000** individuals with increasing trend. As captive breeding efforts on these species are still in development, individuals sold in the market are mostly wild-caught. Which of the following turtles would you most prefer to purchase? [Single choice]

您想要选购一只宠物龟，现有下列几种**淡水龟龟苗**供您选择。它们的野外种群数量均小于**2000**，数量正在**上升**，但人工繁育技术未成熟，市面上以**野生个体为主**。您更倾向于购买下列哪一只龟？[单选题]


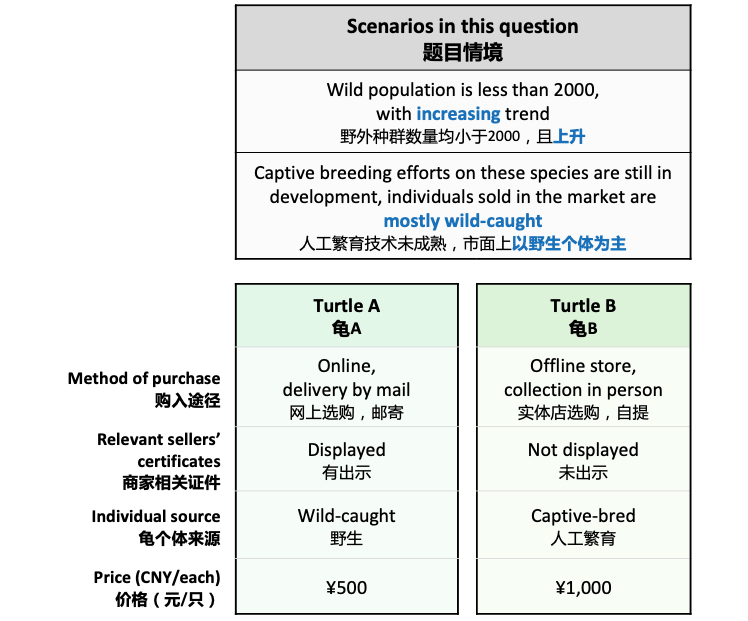


| ○Turtle A龟种A |
| --- |
| ○Turtle B龟种B |
| ○Neither of the turtles above would I like to purchase以上龟种我都不想购买 |

37. **Please imagine the following scenarios:** 请您假设以下情景：

You would like to purchase a pet turtle, and have found the following types of **juvenile freshwater turtles** to choose from. At present, the wild population of these species is less than **2000** individuals with stable population. Captive breeding efforts on these species are developed, and abundant captive-bred individuals are sold in the market. Which of the following turtles would you most prefer to purchase? [Single choice]

您想要选购一只宠物龟，现有下列几种**淡水龟龟苗**供您选择。它们的野外种群数量均小于**2000**，种群数量**稳定**，且人工繁育技术成熟，市面上有**大量人工繁育个体**。您更倾向于购买下列哪一只龟？[单选题]


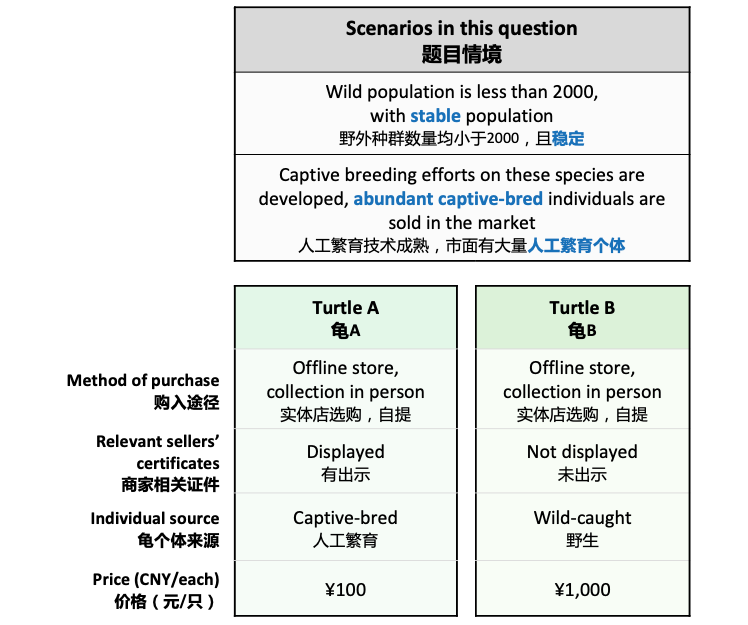


| ○Turtle A龟种A |
| --- |
| ○Turtle B龟种B |
| ○Neither of the turtles above would I like to purchase以上龟种我都不想购买 |

**Subsets 3: Q38-Q43**

**第一组：38-43**

38. **Please imagine the following scenarios:** 请您假设以下情景：

You would like to purchase a pet turtle, and have found the following types of **juvenile freshwater turtles** to choose from. At present, the wild population of these species is less than **2000** individuals with decreasing trend. As captive breeding efforts on these species are still in development, individuals sold in the market are mostly wild-caught. Which of the following turtles would you most prefer to purchase? [Single choice]

您想要选购一只宠物龟，现有下列几种**淡水龟龟苗**供您选择。它们的野外种群数量均小于**2000**，数量仍在**下降**，且人工繁育技术未成熟，市面上以**野生个体为主**。您更倾向于购买下列哪一只龟？[单选题]


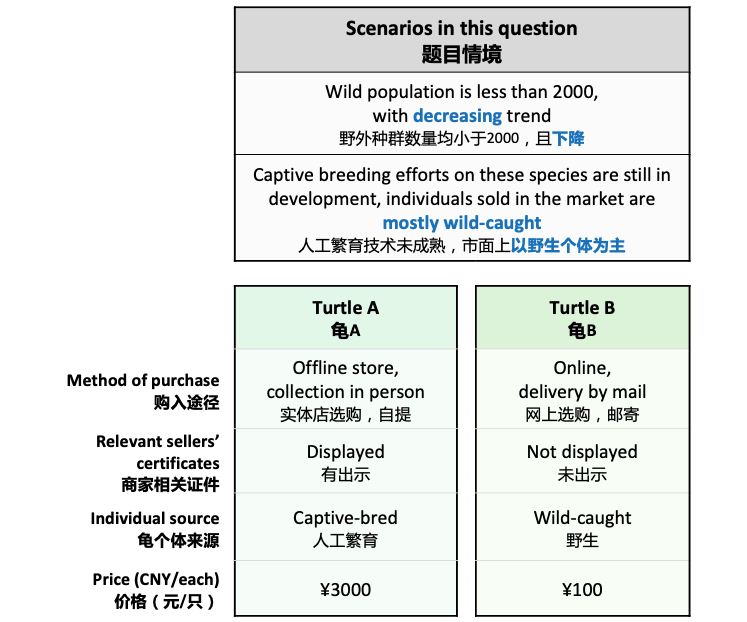


| ○Turtle A龟种A |
| --- |
| ○Turtle B龟种B |
| ○Neither of the turtles above would I like to purchase以上龟种我都不想购买 |

39. **Please imagine the following scenarios:** 请您假设以下情景：

You would like to purchase a pet turtle, and have found the following types of **juvenile freshwater turtles** to choose from. At present, the wild population of these species is less than **2000** individuals with decreasing trend. Captive breeding efforts on these species are developed, and abundant captive-bred individuals are sold in the market. Which of the following turtles would you most prefer to purchase? [Single choice]

您想要选购一只宠物龟，现有下列几种**淡水龟龟苗**供您选择。它们的野外种群数量均小于**2000**，数量仍在**下降**，但人工繁育技术成熟，市面上有**大量人工繁育个体**。您更倾向于购买下列哪一只龟？


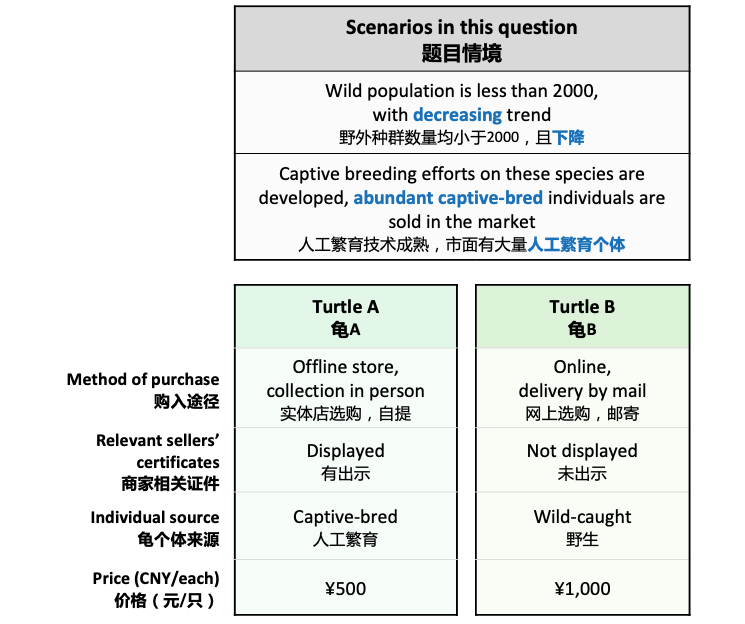


| ○Turtle A龟种A |
| --- |
| ○Turtle B龟种B |
| ○Neither of the turtles above would I like to purchase以上龟种我都不想购买 |

40. **Please imagine the following scenarios:** 请您假设以下情景：

You would like to purchase a pet turtle, and have found the following types of **juvenile freshwater turtles** to choose from. At present, the wild population of these species is less than **2000** individuals with stable population. Captive breeding efforts on these species are developed, and abundant captive-bred individuals are sold in the market. Which of the following turtles would you most prefer to purchase? [Single choice]

您想要选购一只宠物龟，现有下列几种**淡水龟龟苗**供您选择。它们的野外种群数量均小于**2000**，数量**稳定**，但人工繁育技术成熟，市面上有**大量人工繁育个体**。您更倾向于购买下列哪一只龟？


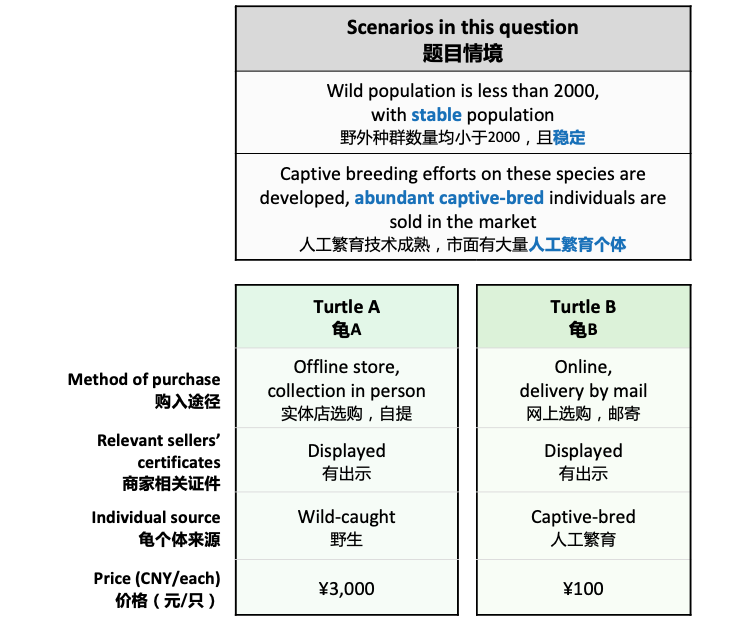


| ○Turtle A龟种A |
| --- |
| ○Turtle B龟种B |
| ○Neither of the turtles above would I like to purchase以上龟种我都不想购买 |

41. **Please imagine the following scenarios:** 请您假设以下情景：

You would like to purchase a pet turtle, and have found the following types of **juvenile freshwater turtles** to choose from. At present, the wild population of these species is less than **2000** individuals with stable population. Captive breeding efforts on these species are developed, and abundant captive-bred individuals are sold in the market. Which of the following turtles would you most prefer to purchase? [Single choice]

您想要选购一只宠物龟，现有下列几种**淡水龟龟苗**供您选择。它们的野外种群数量均小于**2000**，数量**稳定**，且人工繁育技术成熟，市面上有**大量人工繁育个体**。您更倾向于购买下列哪一只龟？[单选题]


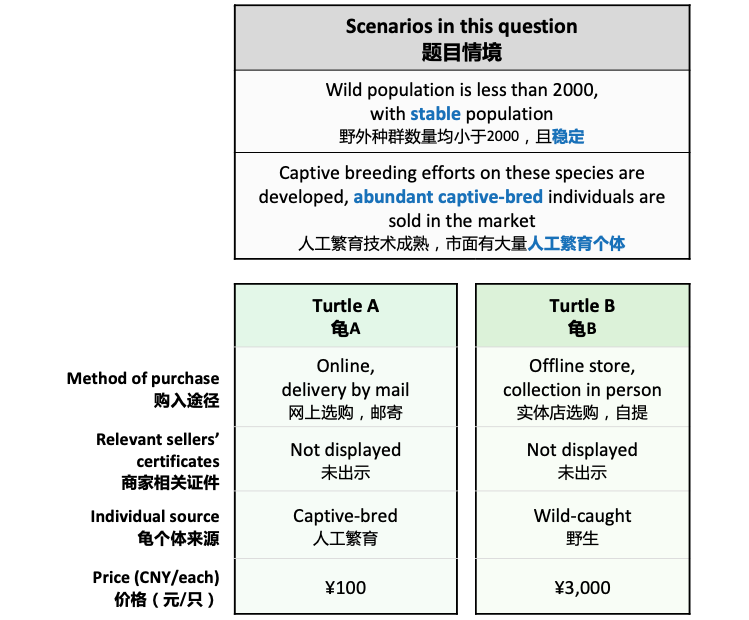


| ○Turtle A龟种A |
| --- |
| ○Turtle B龟种B |
| ○Neither of the turtles above would I like to purchase以上龟种我都不想购买 |

42. **Please imagine the following scenarios:** 请您假设以下情景：

You would like to purchase a pet turtle, and have found the following types of **juvenile freshwater turtles** to choose from. At present, the wild population of these species is less than **2000** individuals with increasing trend. As captive breeding efforts on these species are still in development, individuals sold in the market are mostly wild-caught. Which of the following turtles would you most prefer to purchase? [Single choice]

您想要选购一只宠物龟，现有下列几种**淡水龟龟苗**供您选择。它们的野外种群数量均小于**2000**，数量正在**上升**，但人工繁育技术未成熟，市面上以**野生个体为主**。您更倾向于购买下列哪一只龟？[单选题]


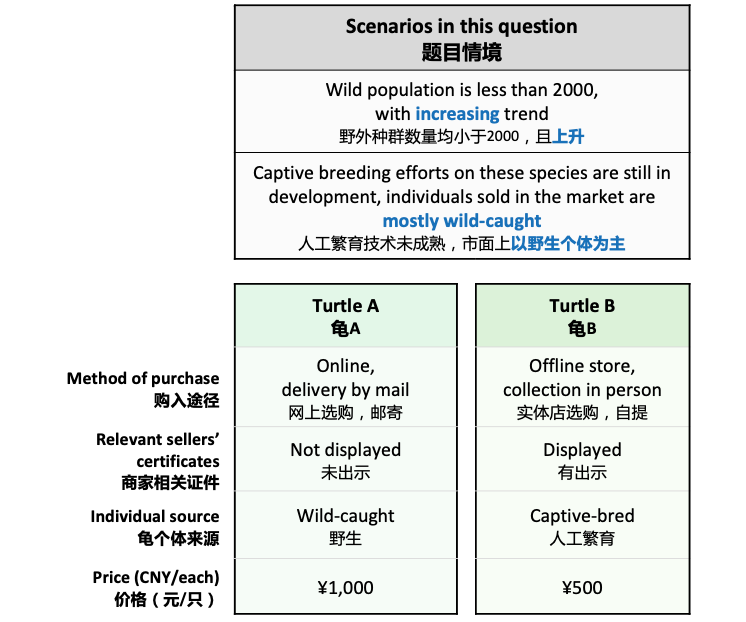


| ○Turtle A龟种A |
| --- |
| ○Turtle B龟种B |
| ○Neither of the turtles above would I like to purchase以上龟种我都不想购买 |

43. **Please imagine the following scenarios:** 请您假设以下情景：

You would like to purchase a pet turtle, and have found the following types of **juvenile freshwater turtles** to choose from. At present, the wild population of these species is less than **2000** individuals with decreasing trend. Captive breeding efforts on these species are developed, and abundant captive-bred individuals are sold in the market. Which of the following turtles would you most prefer to purchase? [Single choice]

您想要选购一只宠物龟，现有下列几种**淡水龟龟苗**供您选择。它们的野外种群数量均小于**2000**，数量仍在**下降**，但人工繁育技术成熟，市面上有**大量人工繁育个体**。您更倾向于购买下列哪一只龟？[单选题]


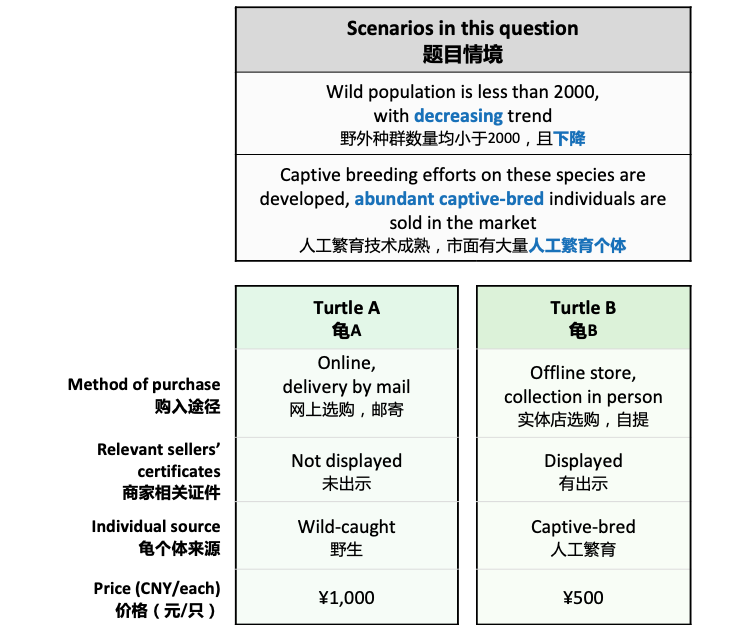


| ○Turtle A龟种A |
| --- |
| ○Turtle B龟种B |
| ○Neither of the turtles above would I like to purchase以上龟种我都不想购买 |

44. There is no right or wrong answer to the questions in the following section, in order to understand the current state of public perception, please answer according to your actual situation. [Single Choice] *

以下部分的问题答案并无对错之分，为了了解目前公众真实的认知现状，请根据您的实际情况回答。[单选题]

○I understand 我已知晓

45. Which of the following turtle species do you believe could be legally sold within China? [Matrix single-choice]

您认为在国内以下哪些龟能够被合法售卖？[矩阵单选题]

| Turtle species | Wild individual  野生个体 | Captive-bred individual  人工繁育个体 | Both types can be sold legally  都可以 | Neither types can be sold legally  都不可以 | I have no idea  我不知道 |
| --- | --- | --- | --- | --- | --- |
| Indochinese box turtle (*Cuora galbinifrons / Cuora bourreti / Cuora picturata*)  黄额闭壳龟 | □ | □ | □ | □ |  |
| Keeled box turtle (*Cuora mouhotii*)  锯缘闭壳龟 | □ | □ | □ | □ |  |
| Southeast Asian box turtle (*Cuora amboinensis*)  马来闭壳龟 | □ | □ | □ | □ |  |
| Big-headed turtle (*Platysternon megacephalum*)  鹰嘴龟 | □ | □ | □ | □ |  |
| Snapping turtle (incl. *Macroclemys temminckii / Chelydra serpentina*)  鳄龟 | □ | □ | □ | □ |  |
| Stink-pot turtle  麝香龟 | □ | □ | □ | □ |  |
| Red-eared slider  巴西龟 | □ | □ | □ | □ |  |
| Other turtle species  其他龟 | □ | □ | □ | □ |  |

46. For pet turtles that require certificates to be sold, what certificates do you think are required for a seller to be qualified? [Multiple Choices]

对于需要证件才能**出售**的宠物淡水龟，您认为具有营业资格的**商家**需要哪些证件？ [多选题]

| □Domestication and Breeding License驯养繁殖许可证（养殖证） |
| --- |
| □Utilization Management Certificate经营利用许可证 |
| □Certificate of Breed引种证明 |
| □Fishing licenses渔业捕捞许可证 |
| □Freshwater Turtles Species Identification Card淡水龟类标识牌 |
| □Not sure我不确定 |

47. Have you applied for any government certificates for keeping pet turtles? [Single Choice]

您是否为了饲养宠物龟办理过任何证件？ [单选题]

| ○Yes办理过 |
| --- |
| ○No没有办理过 |
| ○Prefer not to say不愿透露 |

48. What kind of certifications have you applied for? [Multiple Choices]

您**办理过**哪些证件？ [多选题]

| □Domestication and Breeding License驯养繁殖许可证（养殖证） |
| --- |
| □Utilization Management Certificate经营利用许可证 |
| □Certificate of Breed引种证明 |
| □Fishing licenses渔业捕捞许可证 |
| □Freshwater Turtles Species Identification Card淡水龟类标识牌 |
| □Other _________________其他 |
| □Other people help me with that, I have no idea他人代办，我不知道 |

Rely on option 1 of question 47 依赖于第47题第1个选项

49. For pet turtles that require certificates to be kept, what certificates do you think keepers need? [Multiple Choices]

对于需要证件才能**饲养**的宠物淡水龟，您认为**饲养者**需要哪些证件？ [多选题]

| □Domestication and Breeding License驯养繁殖许可证（养殖证） |
| --- |
| □Utilization Management Certificate经营利用许可证 |
| □Certificate of Breed引种证明 |
| □Fishing licenses渔业捕捞许可证 |
| □Freshwater Turtles Species Identification Card淡水龟类标识牌 |
| □Other _________________其他 |
| □No certifications are required if purchase from a licensed merchant从有证商家处购买则不需要任何证件 |
| □Not sure我不确定 |

Rely on option 2&3 of question 47

50. If you can no longer keep your turtle, which of the following options would you like to try to place your turtle? [Multiple Choices]

如您无法再继续饲养您的宠物龟，您愿意尝试以下哪些方式安置您的宠物龟？ [多选题]

| □Release放生 |
| --- |
| □Resell转卖 |
| □Give it to others赠与他人 |
| □Transfer the turtle to rescue centers or zoo将龟种转交到救护中心或动物园 |
| □Other_________________其他 |
| □Not sure我不确定 |

51. How likely would you report illegal purchases/trade activities[/behaviors] on pet turtles? [Single Choice]

您有多大可能会匿名举报非法的宠物龟买卖行为？ [单选题]

| ○Very unlikely非常不可能 | ○Somewhat unlikely  不太可能 | ○Average  中立 | ○Somewhat likely  有可能 | ○Very likely非常可能 |
| --- | --- | --- | --- | --- |

52. What are the main reasons that may concern or discourage you from pursuing to report? [Multiple Choices]

您不愿意举报的原因可能有哪些？ [多选题]

| □Worried about offending others担心得罪人 |
| --- |
| □complicated to conduct操作复杂 |
| □Received no response following report举报没有回应 |
| □Not relevant to me与自己无关 |
| □Unsure or unclear of how to report不知道如何举报 |
| □Other _________________其他 |

Rely on the options 1&2&3&4&5 of question 51

依赖于第51题第1;2;3;4;5个选项

53. Do you agree the following statements [Likert Matrix] *

|  | Strongly disagree  非常  不同意 | Somewhat disagree  比较  不同意 | Average  一般 | Somewhat agree  比较同意 | Strongly  Agree  非常同意 |
| --- | --- | --- | --- | --- | --- |
| I know exactly what kind of turtle I am keeping  我明确地知道我所饲养的龟是什么种类 | ○ | ○ | ○ | ○ | ○ |
| I can differentiate between wild turtles and captive-bred turtles  我能够辨别野生的龟和人工繁育来源的龟 | ○ | ○ | ○ | ○ | ○ |
| I know which turtle species are endangered  我知道哪些龟种是濒危的物种 | ○ | ○ | ○ | ○ | ○ |
| I know which turtle species are illegal to keep  我知道饲养哪些龟种是非法的 | ○ | ○ | ○ | ○ | ○ |
| I know how or where to report illegal wildlife trade activities  我知道如何举报非法的动物贸易 | ○ | ○ | ○ | ○ | ○ |
| Keeping endangered turtle species as pets is a way to protect species populations  饲养濒危龟种作为宠物是一种保护它们的种群的方式 | ○ | ○ | ○ | ○ | ○ |
| I admire people who have or keep rare turtle species as pets  我羡慕拥有珍稀宠物龟的人 | ○ | ○ | ○ | ○ | ○ |
| Compared to keepers of rare turtle species or varieties, I much rather admire people who can keep common varieties well  与养殖稀有龟种或品种的养龟者相比，我更钦佩那些能够养好常见品种的人 | ○ | ○ | ○ | ○ | ○ |
| Please select "somewhat disagree"  请选择“比较不同意” | ○ | ○ | ○ | ○ | ○ |
| Releasing pet turtles in the wild is good for increasing their wild populations  放生宠物龟对于增加龟种野外种群数量来说是一件好事 | ○ | ○ | ○ | ○ | ○ |
| Most people find it difficult to confirm whether the turtles they purchase are from legal origin  大多数人很难确认自己所购买宠物龟的来源是否合法 | ○ | ○ | ○ | ○ | ○ |
| Pet turtles of legal origin are more expensive to purchase than pet turtles of illegal origin  合法来源的宠物龟比起非法的龟种更昂贵 | ○ | ○ | ○ | ○ | ○ |
| Buying legally-sourced turtles are often very complicated or involves complex procedures  购买合法的龟往往有许多复杂的程序 | ○ | ○ | ○ | ○ | ○ |
| Buying illegally-sourced turtles is very common to see or hear about amongst people  购买非法龟种是一件常见的事 | ○ | ○ | ○ | ○ | ○ |
| Enforcement surrounding the illegal trade of pet turtles is currently very strict  目前关于非法宠物龟贸易的执法非常严格 | ○ | ○ | ○ | ○ | ○ |
| Keeping illegal turtles is not easy to be caught  饲养非法宠物龟不容易被抓 | ○ | ○ | ○ | ○ | ○ |
| The consequences from buying illegal turtles are not serious  饲养非法宠物龟所需要承担的法律后果并不严重 | ○ | ○ | ○ | ○ | ○ |
| Even if the species they want to buy is listed into China’s List of Wildlife under Special State Protection，people will still insist on buying them即使想要购入的龟种被列入了国家重点野生动物保护名录，人们仍然会坚持购买 | ○ | ○ | ○ | ○ | ○ |

54. Please select your gender: [Single Choice]

您的性别 [单选题]

| ○Male男 | ○Female女 | ○Other其他 | ○Prefer not to say不愿透露 |
| --- | --- | --- | --- |

55. Your residence during the latest year is: [Fill in the blank]

您最近一年常住地是: [填空题]

_________________________________

56. What is your current employment status? [Single Choice]

您目前的工作状态是？ [单选题]

| ○Full-time employment全职工作 |
| --- |
| ○Part-time employment兼职工作 |
| ○- Housewife/husband家庭主妇/夫 |
| ○Student学生 |
| ○Retired退休 |
| ○Independent/Freelance个体 |
| ○Other, _________________其他 |

57. What is your current occupation or industry of employment: [Multiple Choice]

您目前所从事的行业： [多选题]

| □Biology/ecology/environmental protection生物 / 生态 / 环保 |
| --- |
| □Medicine and Health医药卫生 |
| □Education教育 |
| □Culture, Sports and entertainment文化体育与娱乐 |
| □Economic/Financial management经济 / 金融管理 |
| □Internet / IT / Computer science互联网 / IT / 计算机 |
| □Industrial / Manufacturing工业 / 制造业 |
| □Construction/Real Estate建筑 / 房地产 |
| □A cadre/civil servant in a government office政府机关干部 / 公务员 |
| □Other, _________________其他 |

Rely on option 1&2 of question 56

依赖于第56题第1;2个选项

58. What is your highest level of education: [Single Choice]

您的最高教育程度是 [单选题]

| ○Elementary school or below小学及以下 |
| --- |
| ○Middle school初中 |
| ○High school and/or vocational school高中或中专 |
| ○Junior college and/or university undergraduate本科或大专 |
| ○University postgraduate masters and above硕士及以上 |

59. What is your annual income? Please select the option that is most fitting to you [Single Choice]

您的**年收入**是？请选出最合适的一项。 [单选题]

| ○Less than ￥10,000 少于1万元 |
| --- |
| ○￥10,000-50,000 1-5万元 |
| ○￥50,000-100,000 5-10万元 |
| ○￥100,000-150,000 10-15万 |
| ○￥150,000-200,000 15-20万 |
| ○￥200,000 and above 20万元以上 |

60. Where did you see this survey? [Single Choice]

您是从哪里看到这份问卷的？ [单选题]

○Tieba _________________贴吧

Please verify 请注明

○Douban ___ ____________豆瓣

Please verify 请注明

○Transfer from friends 朋友转发

61. Do you anything else want to share with us? [Fill in the blank]

您还有什么想要和我们说的吗 [填空题]

________________________________
